# Supplementary material for: Tumor organoids modeling reveals timed responses and interplay of radiotherapy and chemotherapy in pancreatic cancer
Source: Radiother Oncol. Author manuscript; Available in PMC 2026 May 30. (PMC13221962; doi:10.1016/j.radonc.2026.111470)
Supplement: 1 [file NIHMS2179288-supplement-1.docx]

**Supplementary Material: Tumor Organoids Modeling Reveals Timed Responses and Interplay of Radiotherapy and Chemotherapy in Pancreatic Cancer**


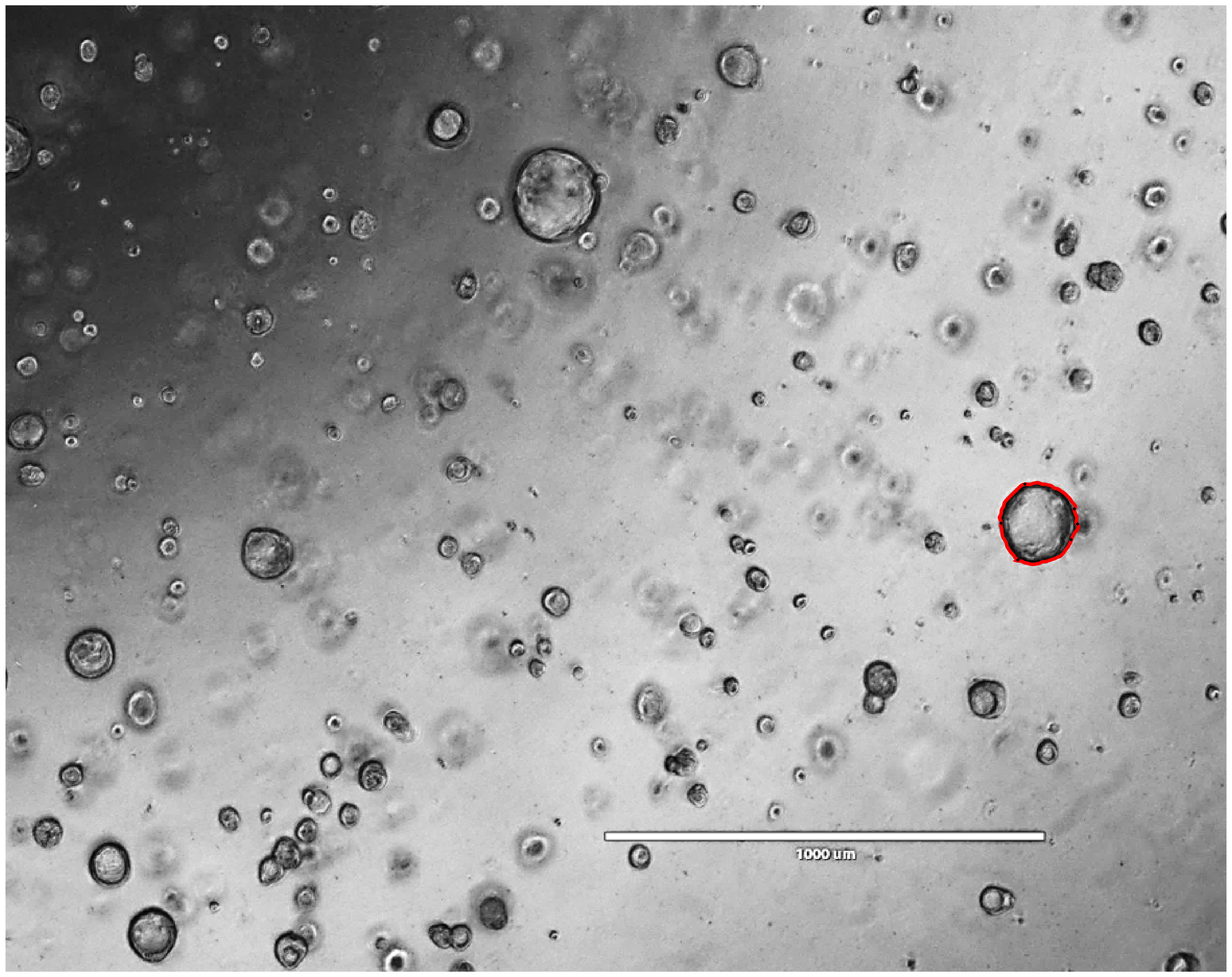

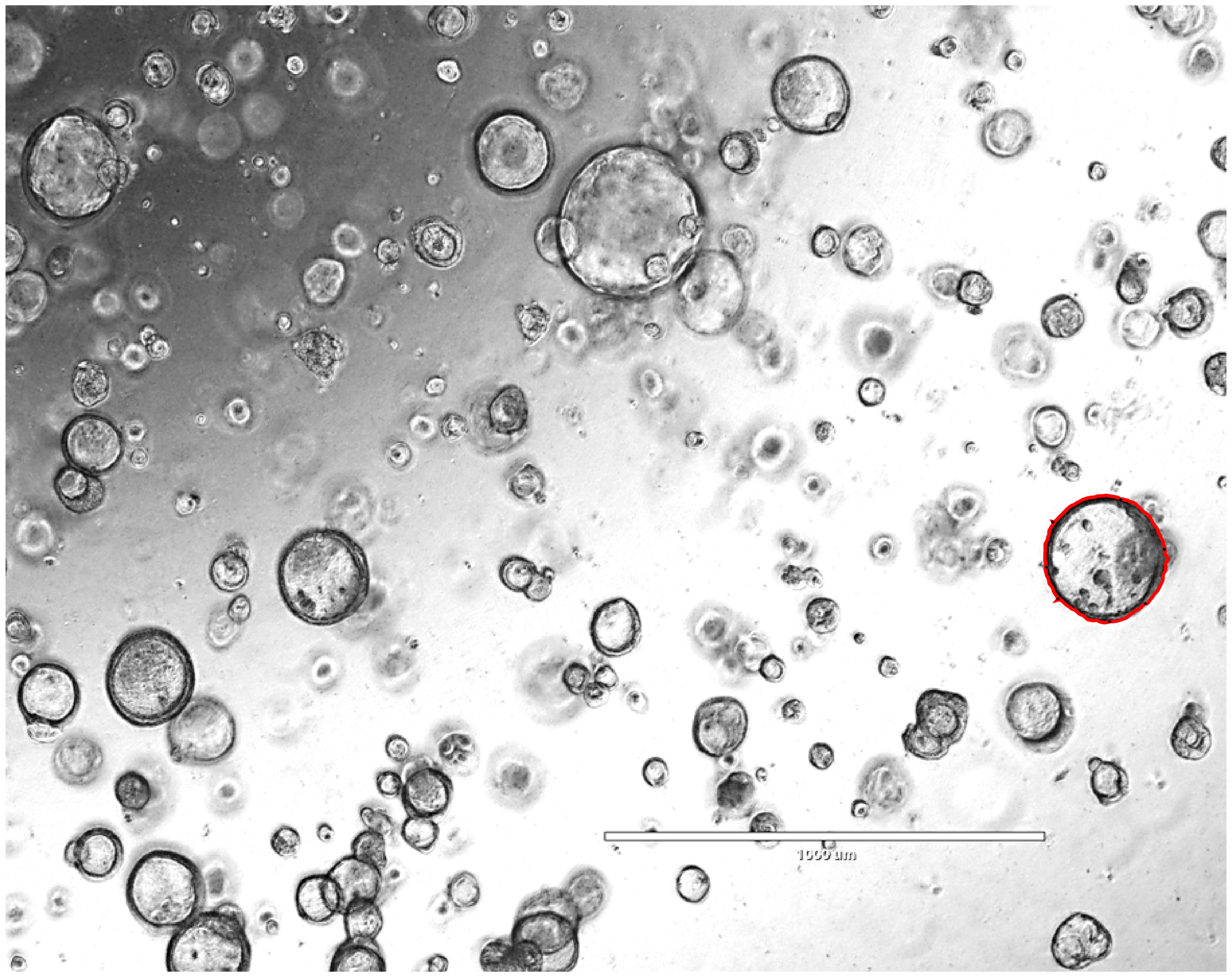


Day 0 Day 2


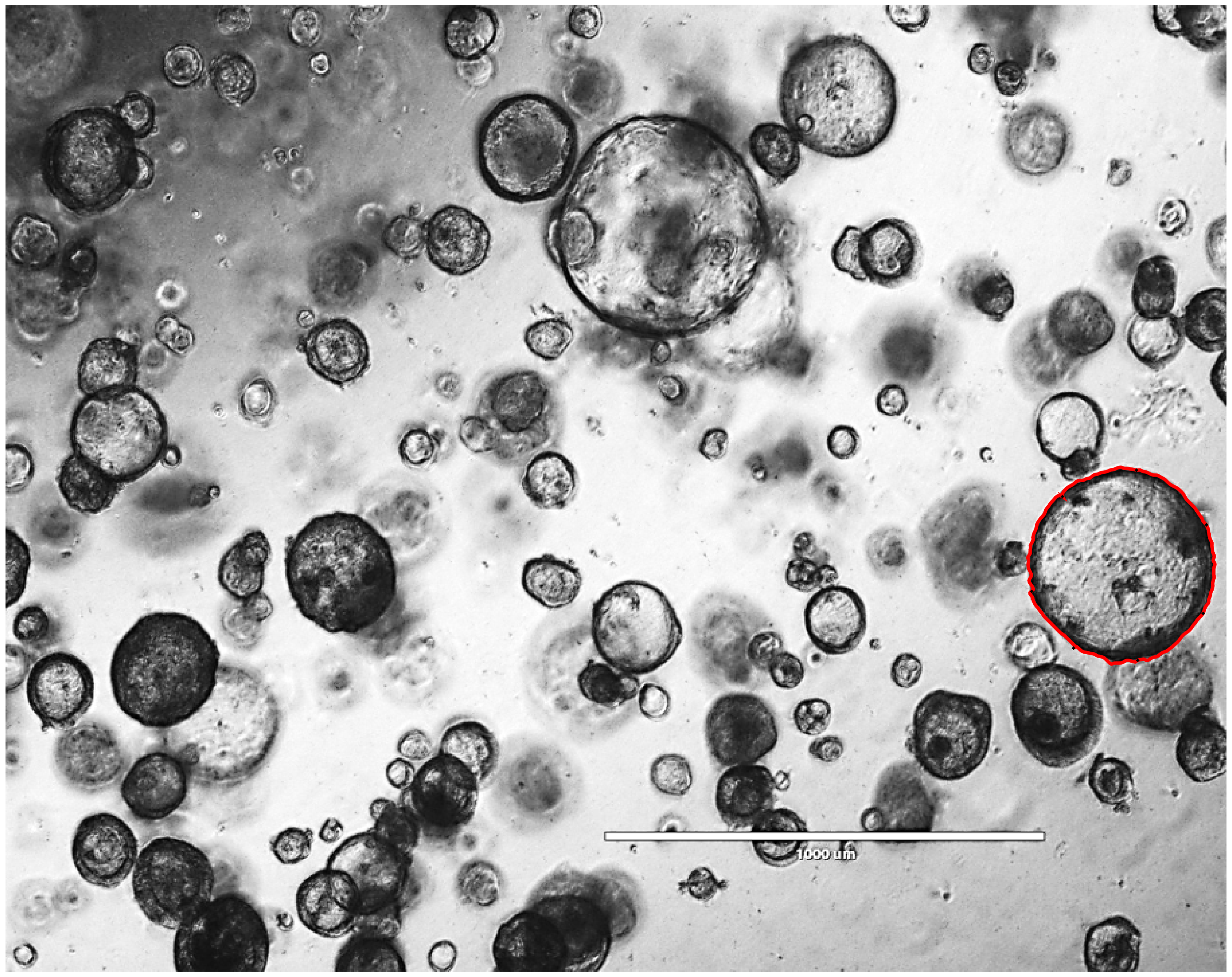

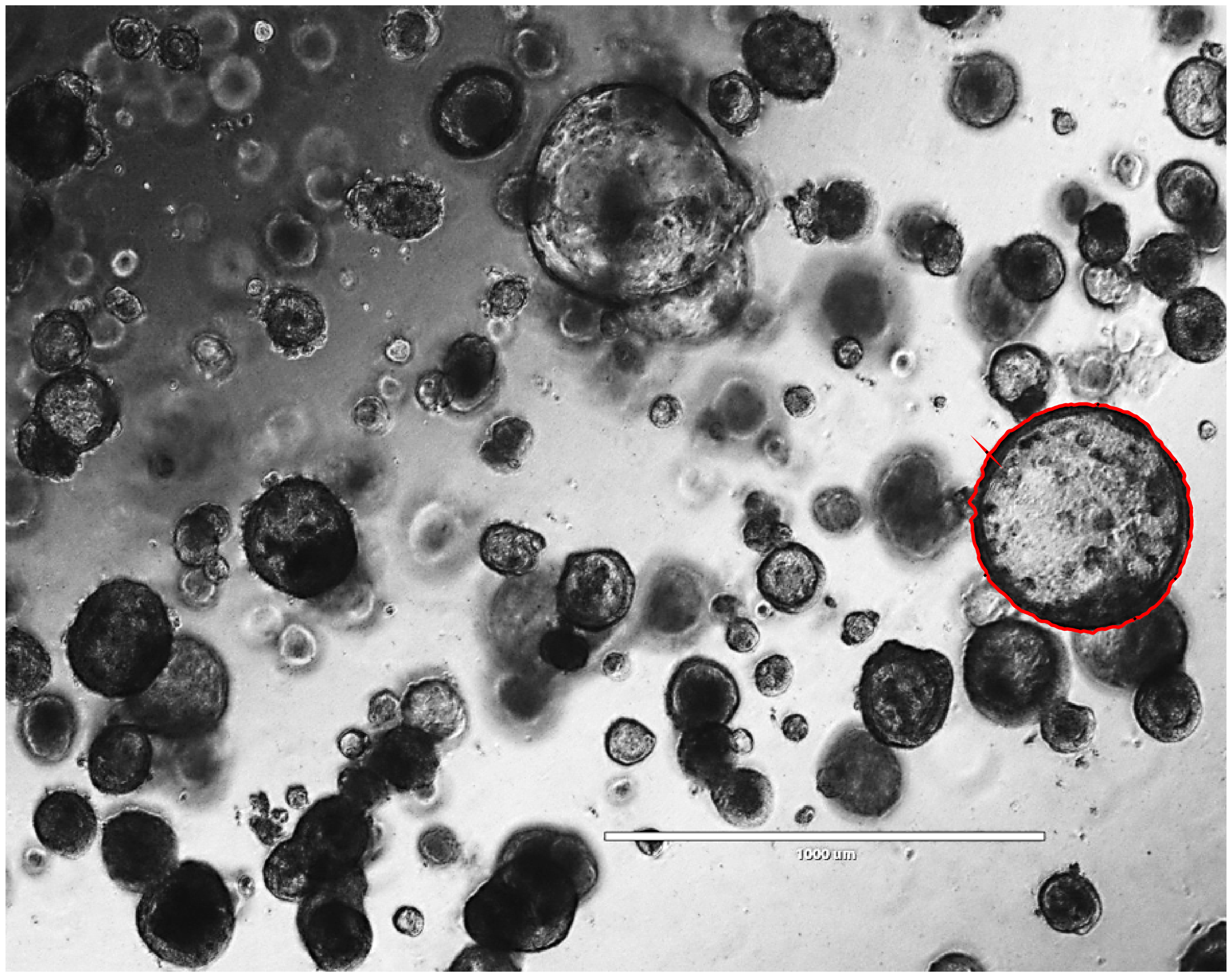


Day 4 Day 6

**Figure S1.** Brightfield image samples of organoids #8510 on days 0, 2, 4, and 6 after radiation of 4 Gy. The typical organoid size was determined by area *S* enclosed by the red circle, that is, the organoid diameter is calculated by $D={2\left( \frac{S}{\pi} \right)}^{1/2}$. Scale bar = 1000 μm.


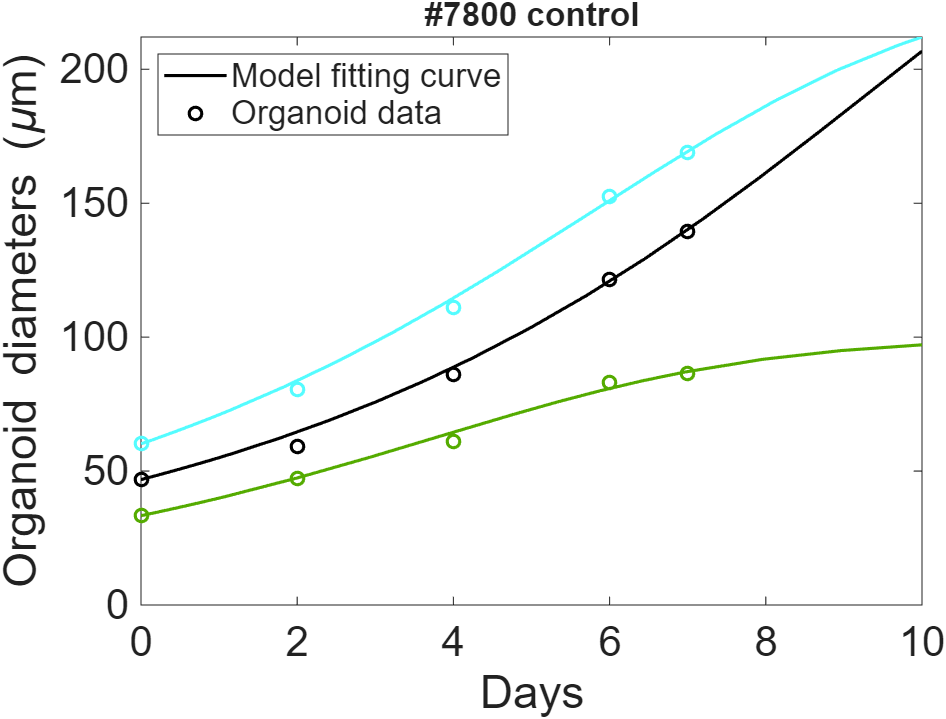

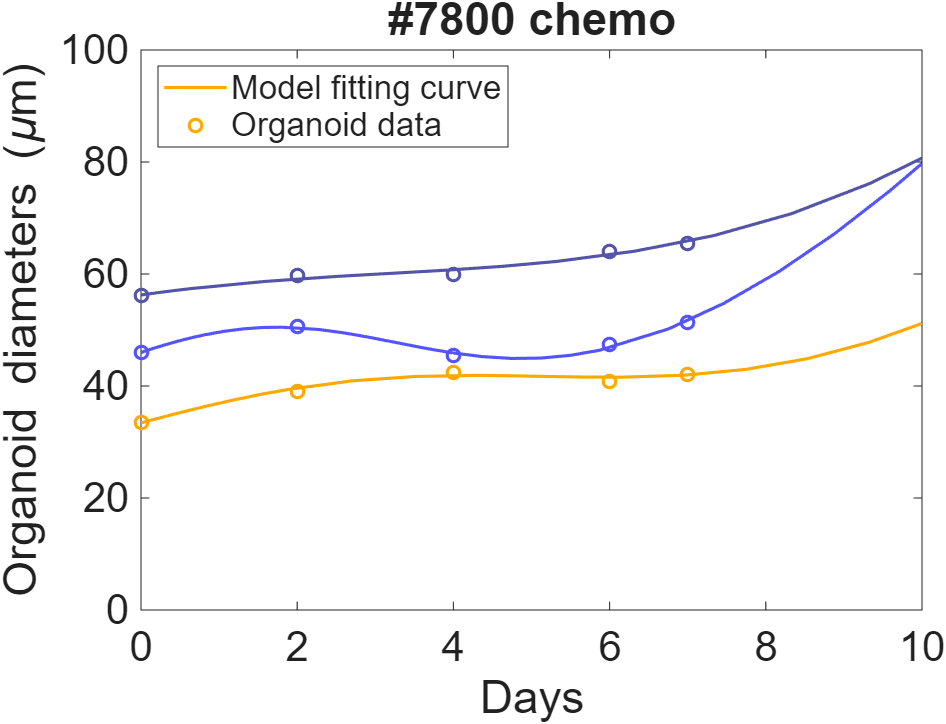


**A B**


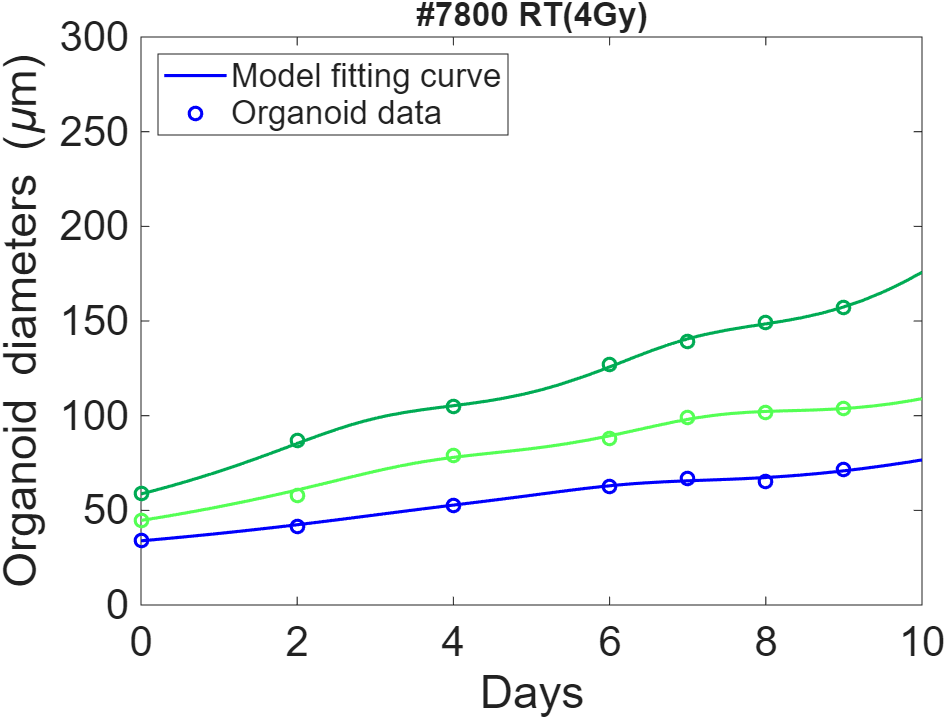

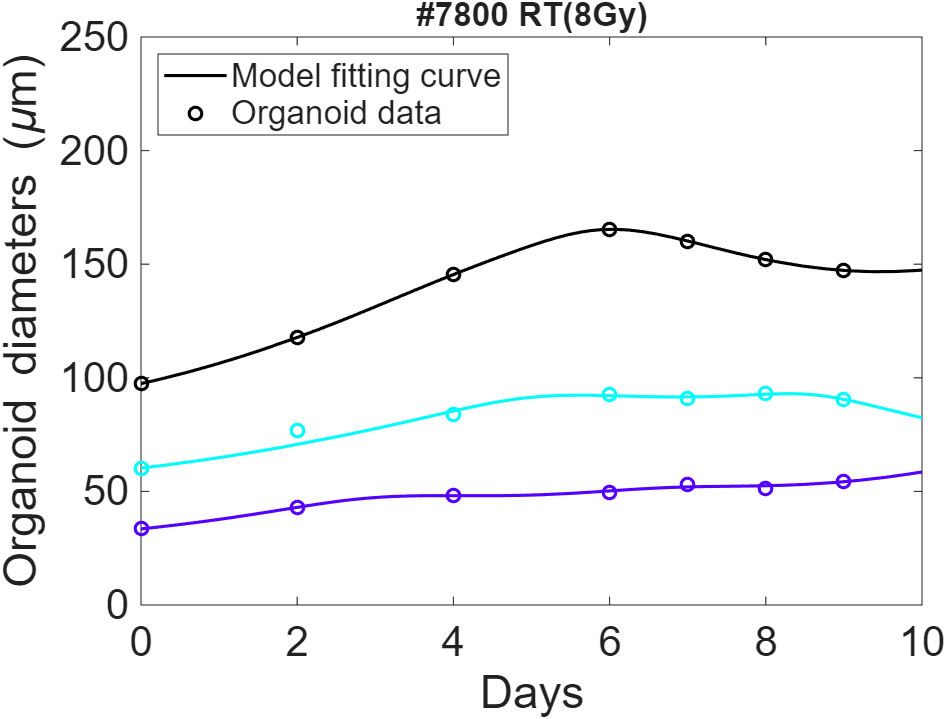


**C D**


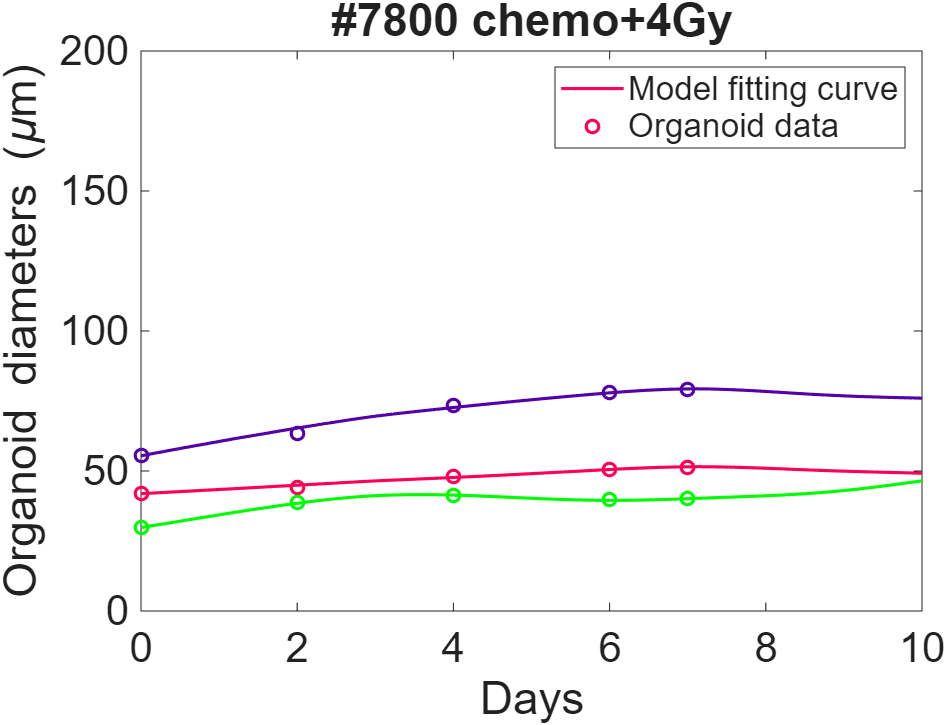

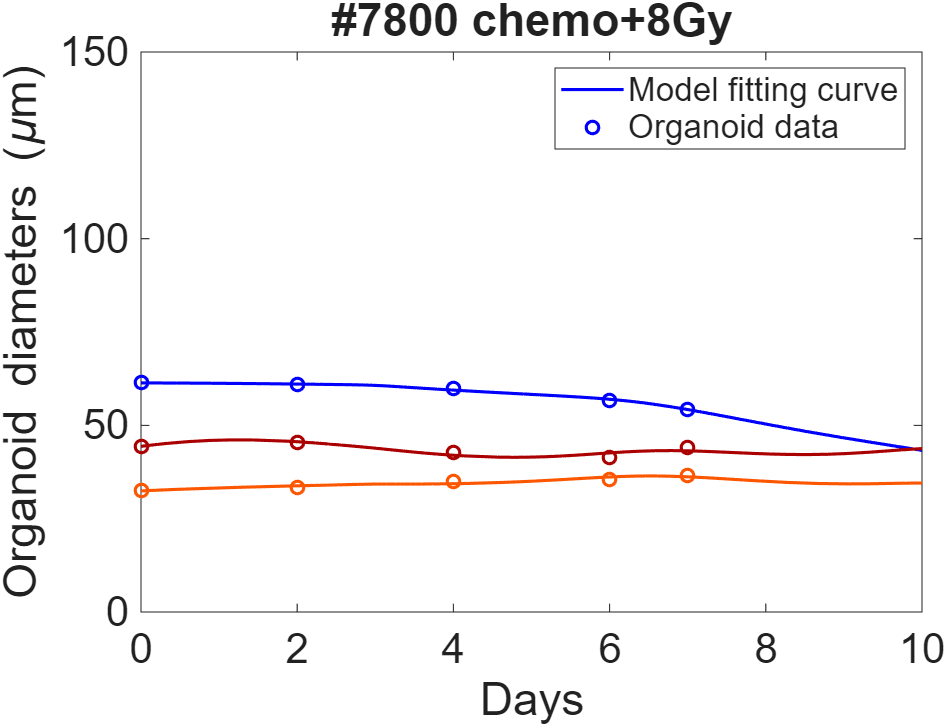


**E F**

**Figure S2.** Three examples of fitting curves for organoids #7800 in each group for clearer visualization.


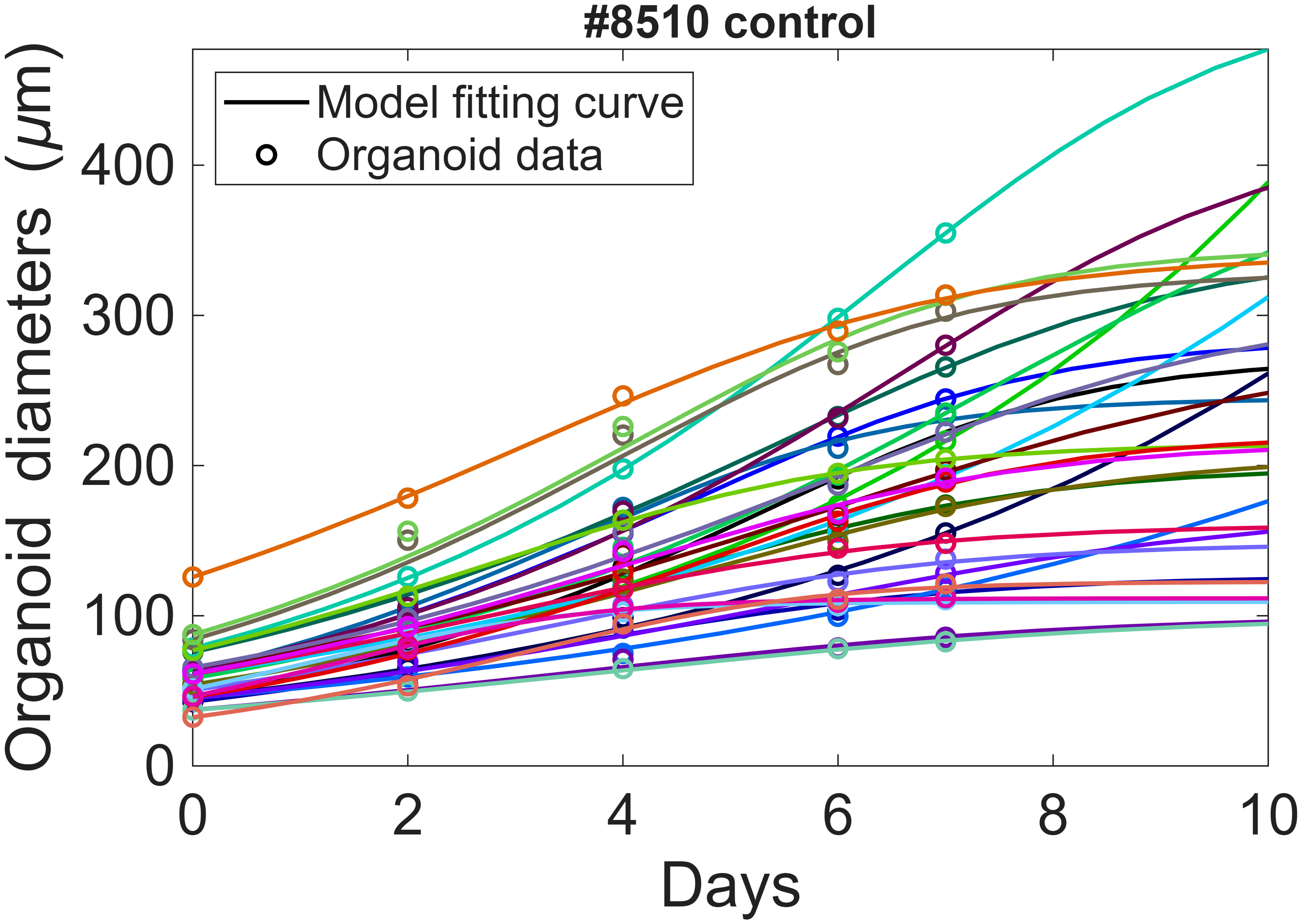

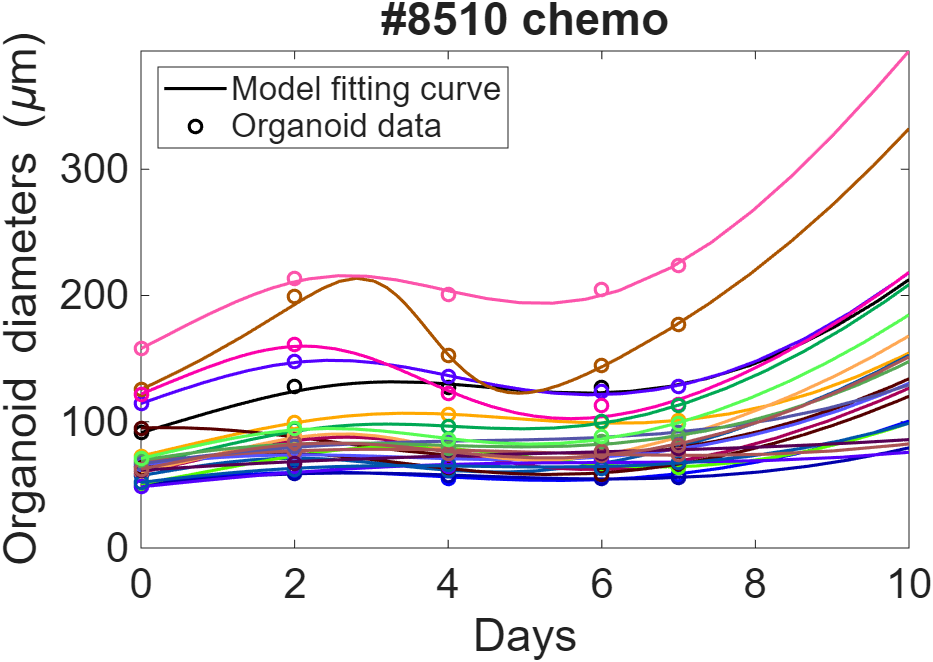


**A B**


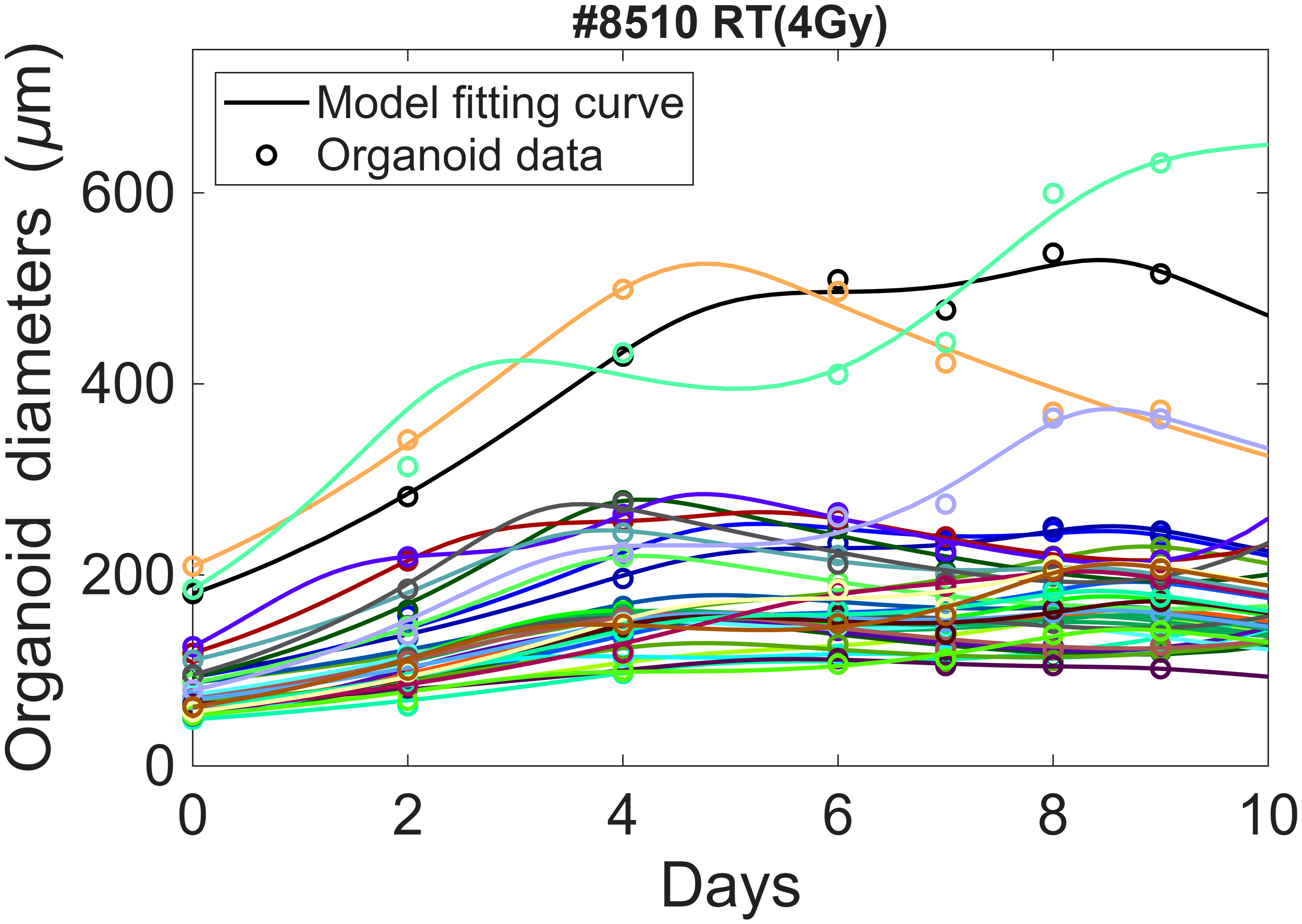

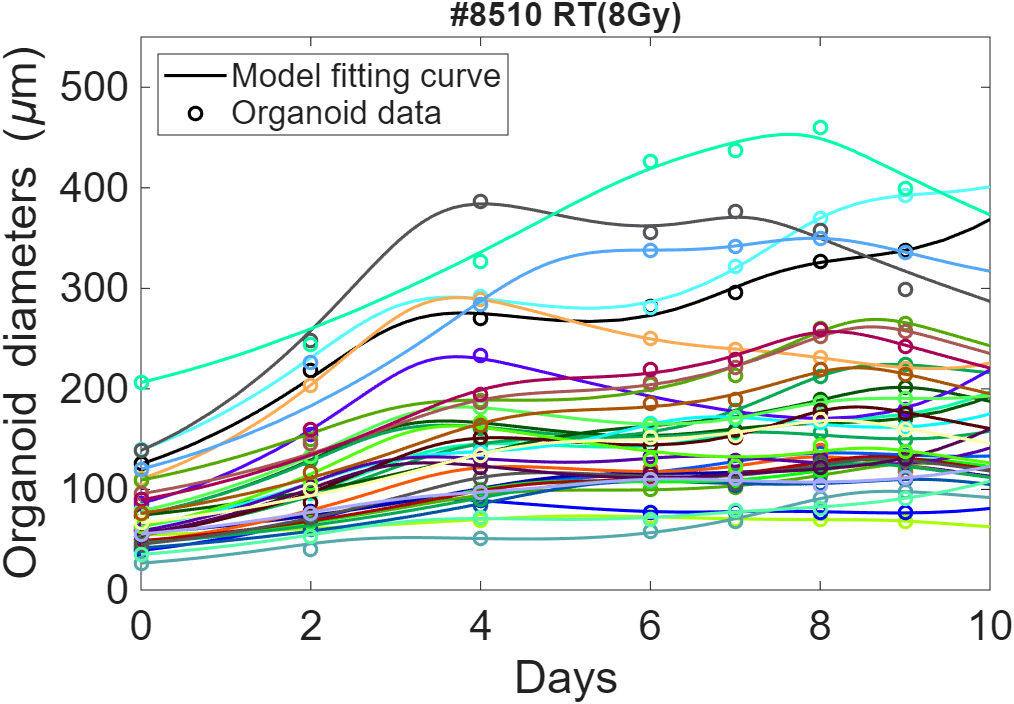


**C D**


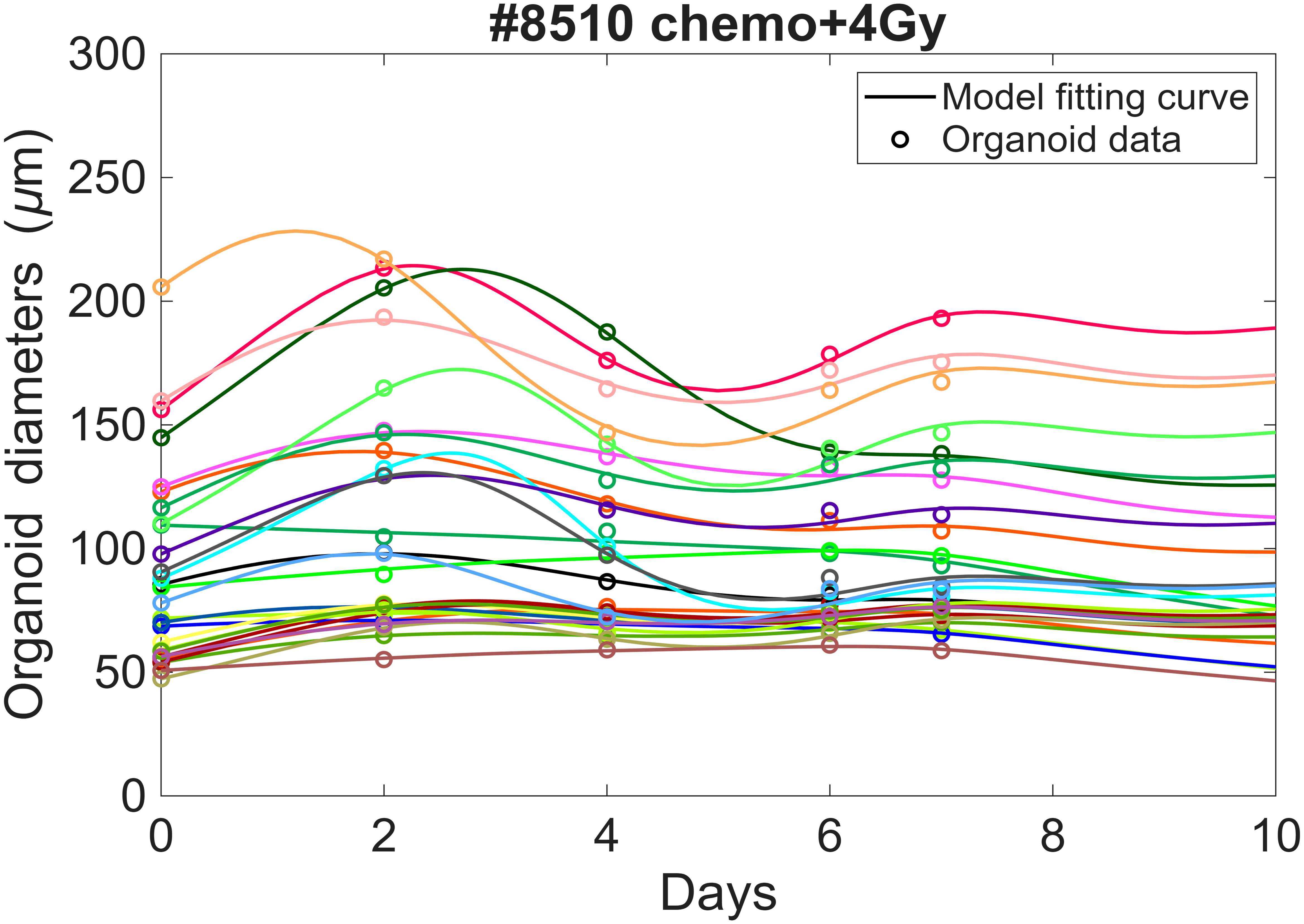

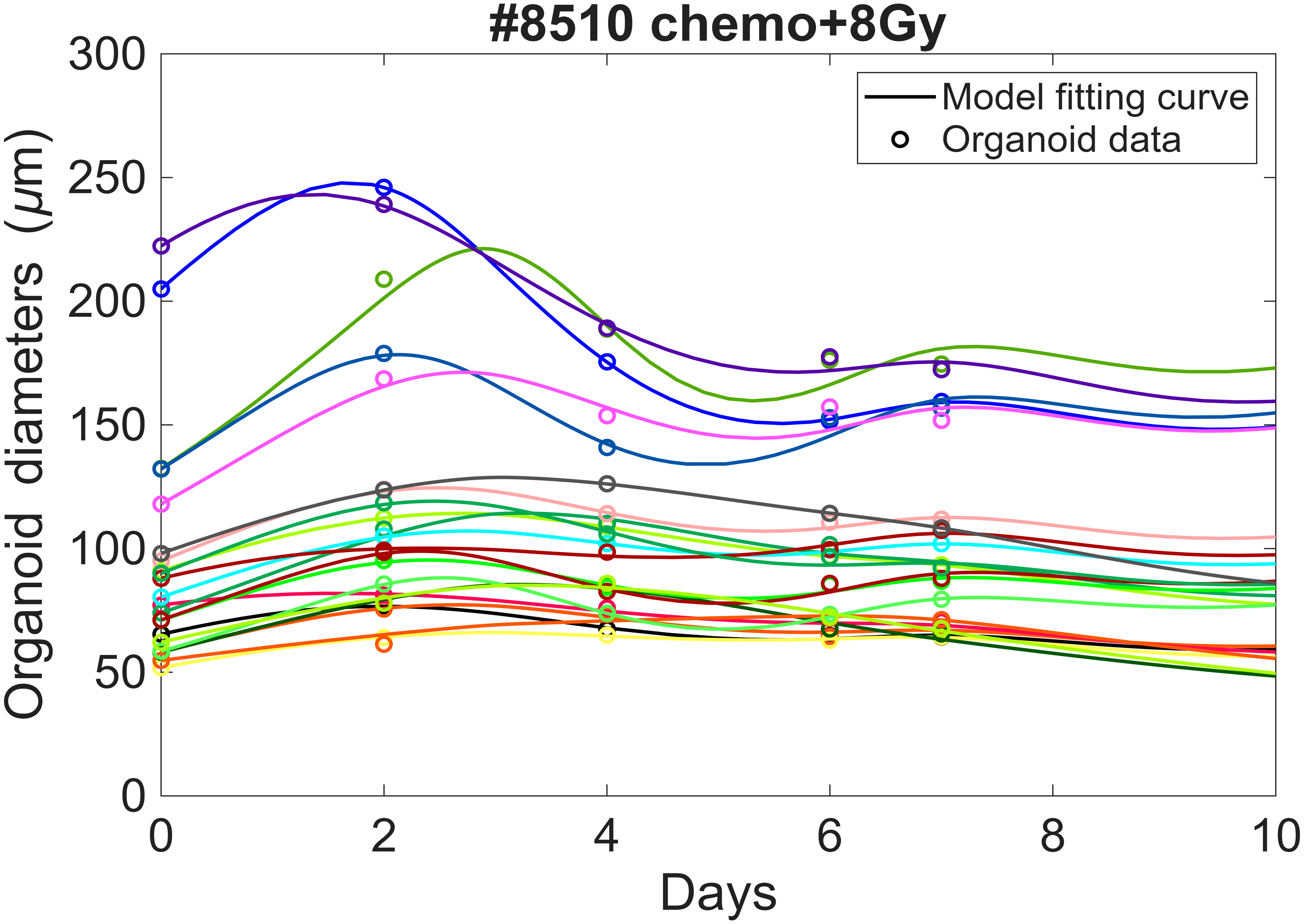


**E F**

**Figure S3.** Model curve fittings of organoids #8510 in each group. Solid lines are fitted curves, and circles of the same color represent data from one organoid collected on corresponding days. There are 30, 24, 37, 37, 28, and 22 organoid size data collected in the control, chemotherapy, radiotherapy with 4 Gy, radiotherapy with 8 Gy, chemoradiotherapy with 4 Gy, and chemoradiotherapy with 8 Gy groups, respectively. The data points were taken up to 7 or 9 days for each group.


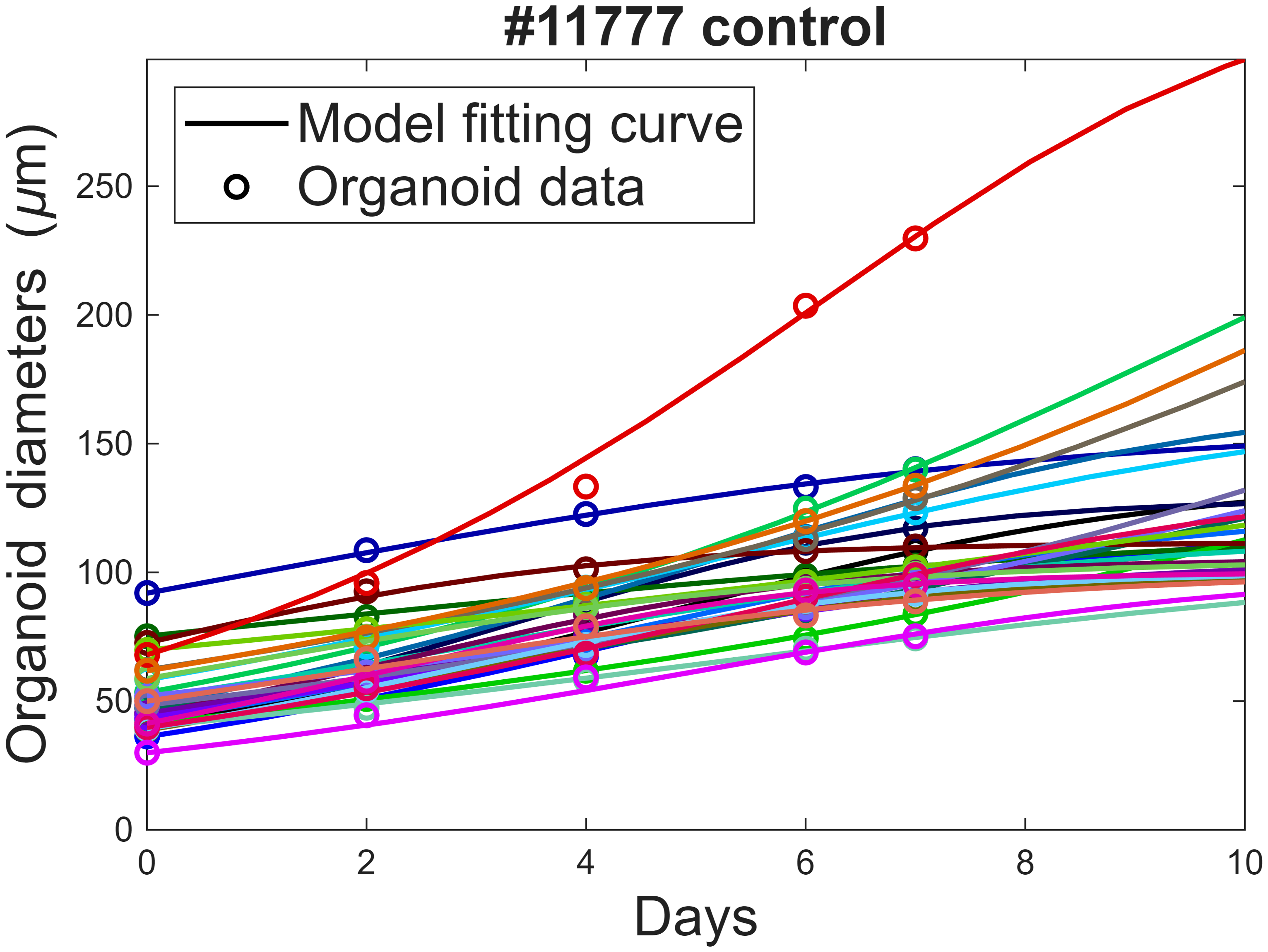

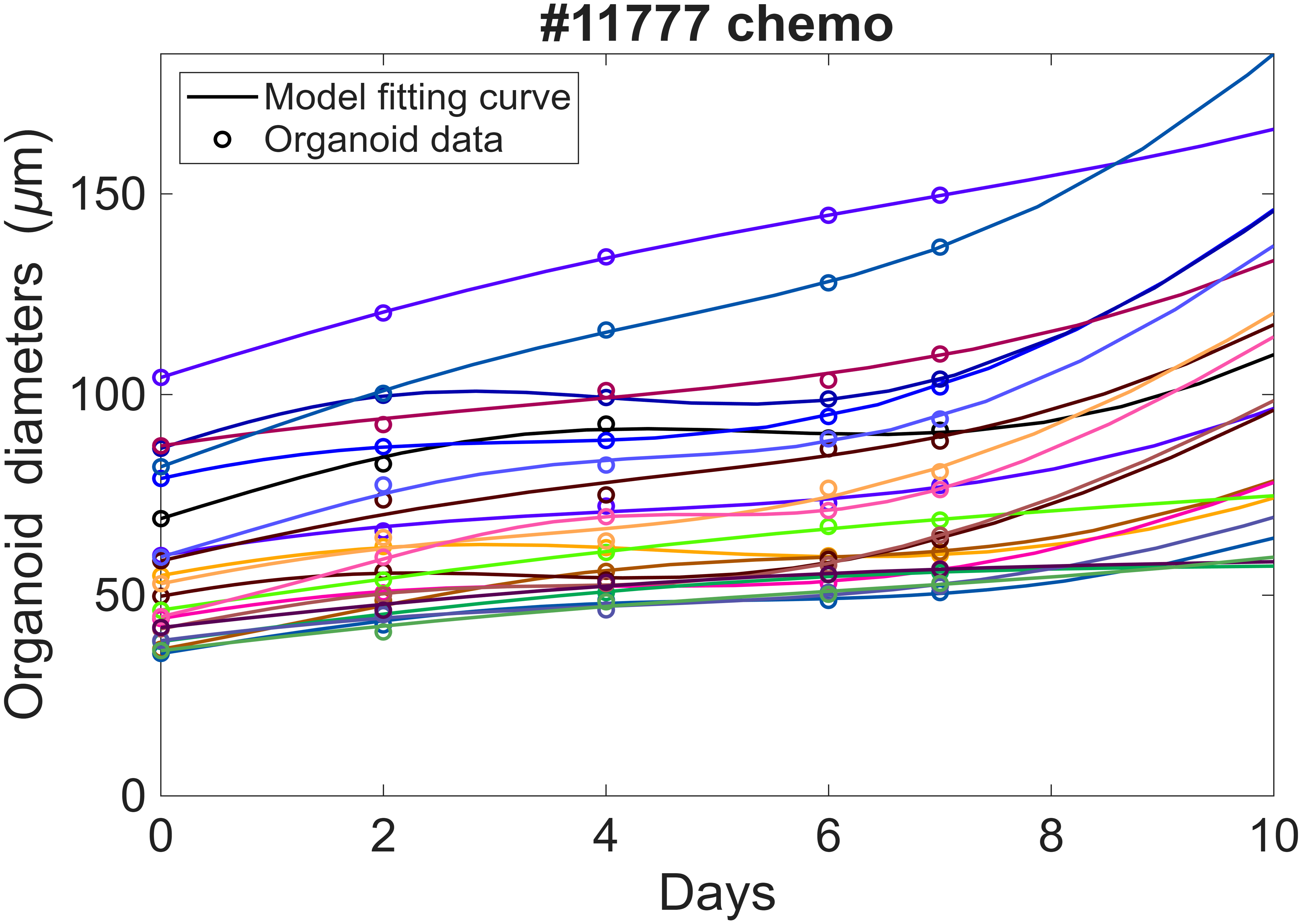


**A B**


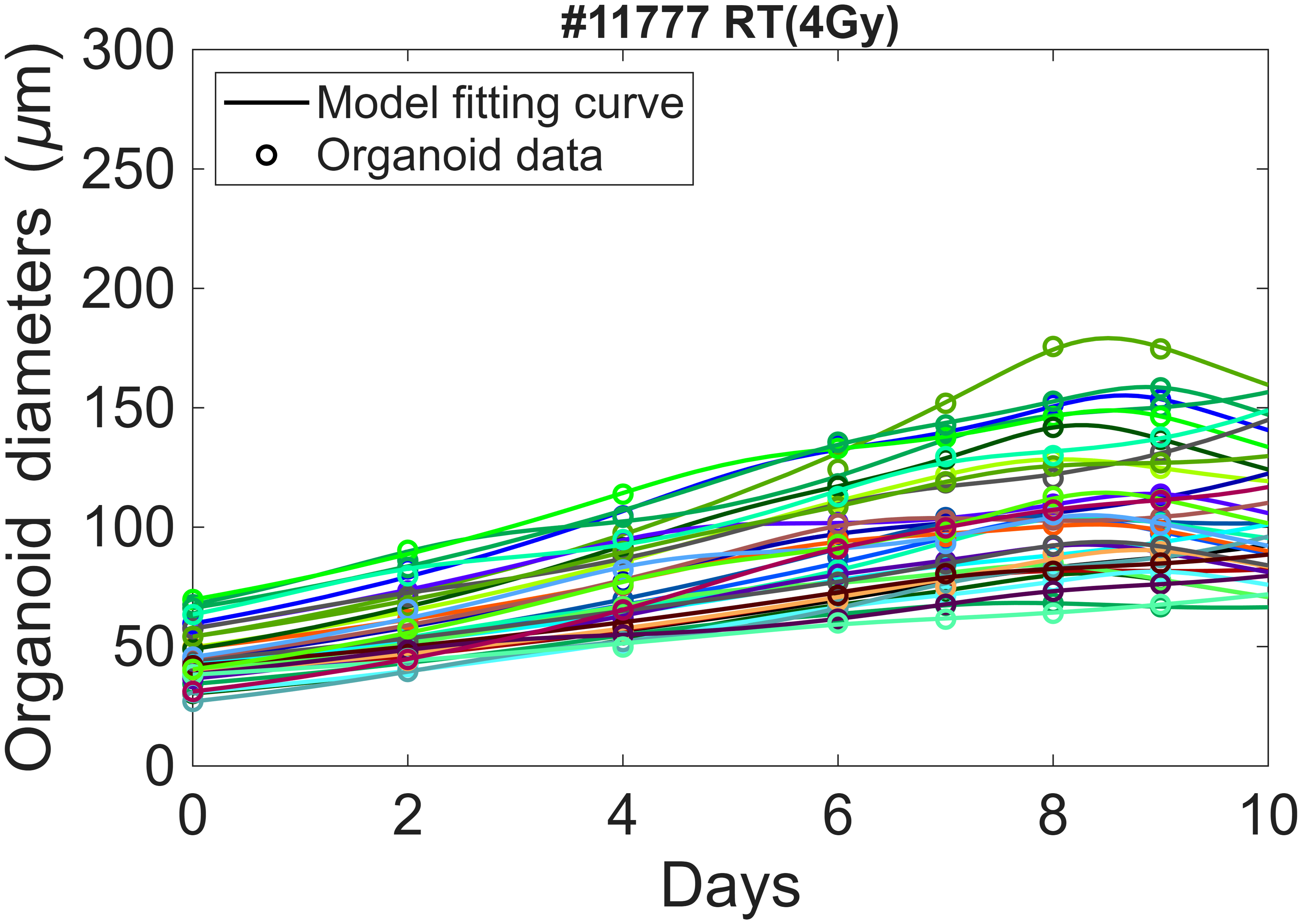

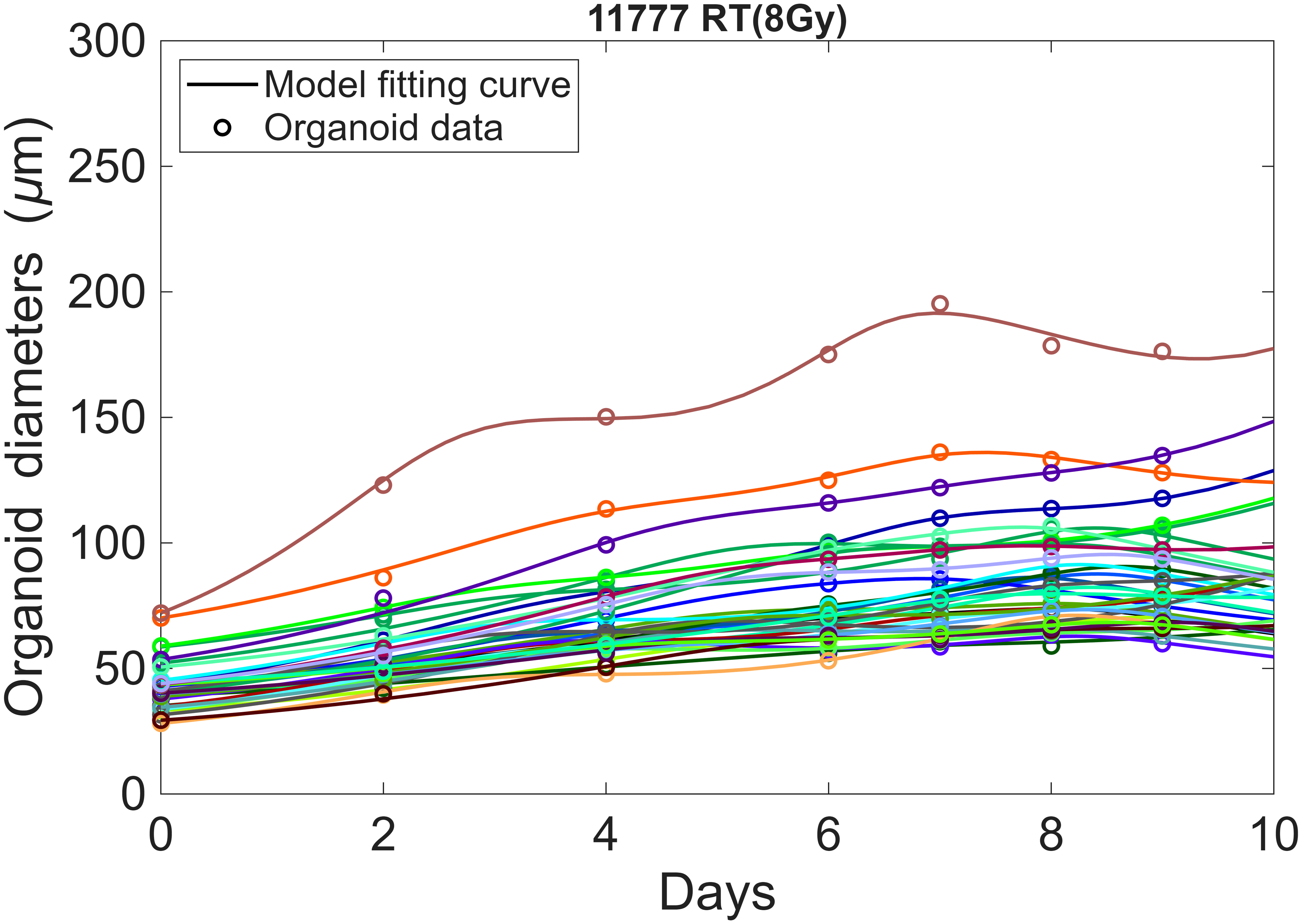


**C D**


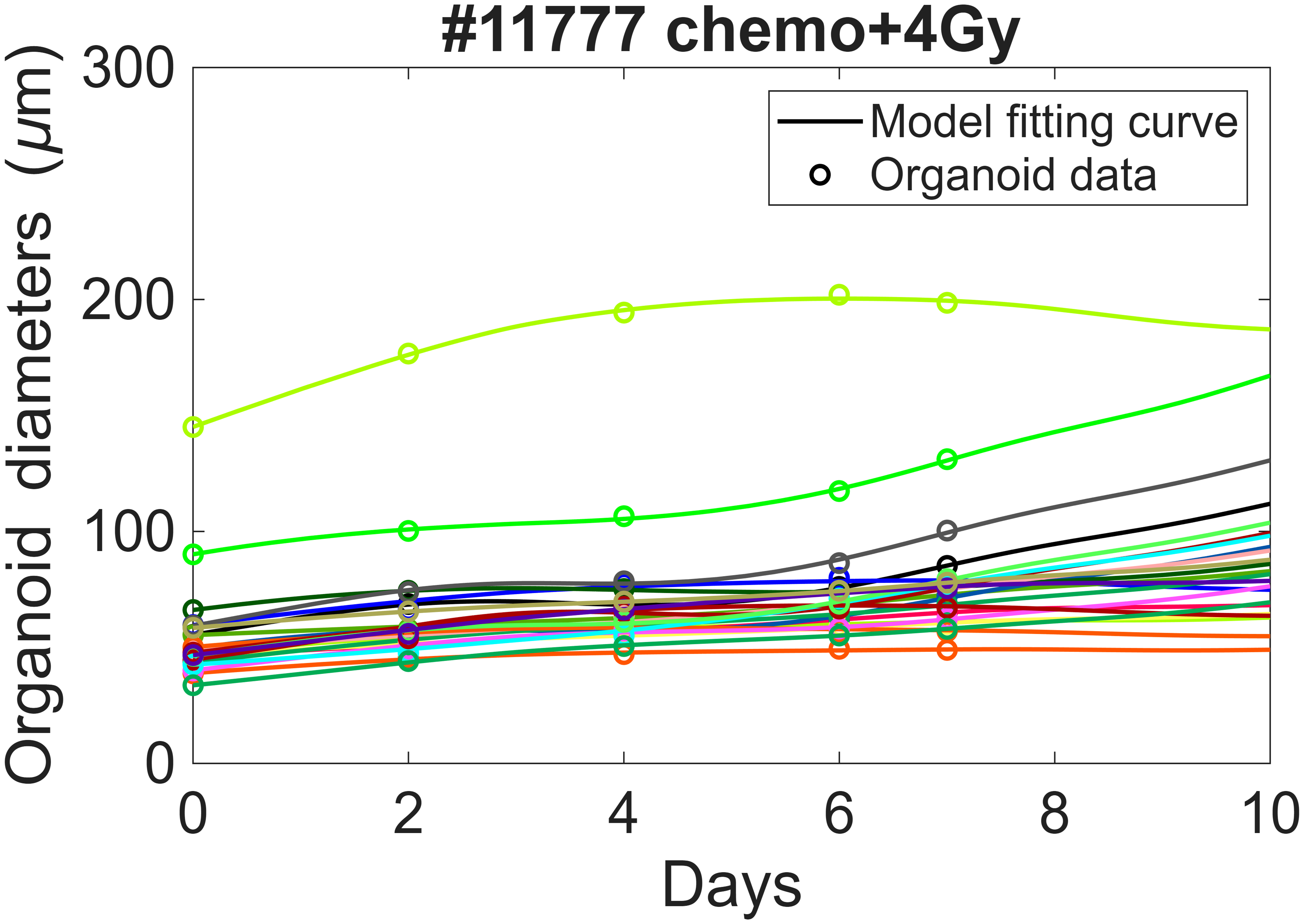

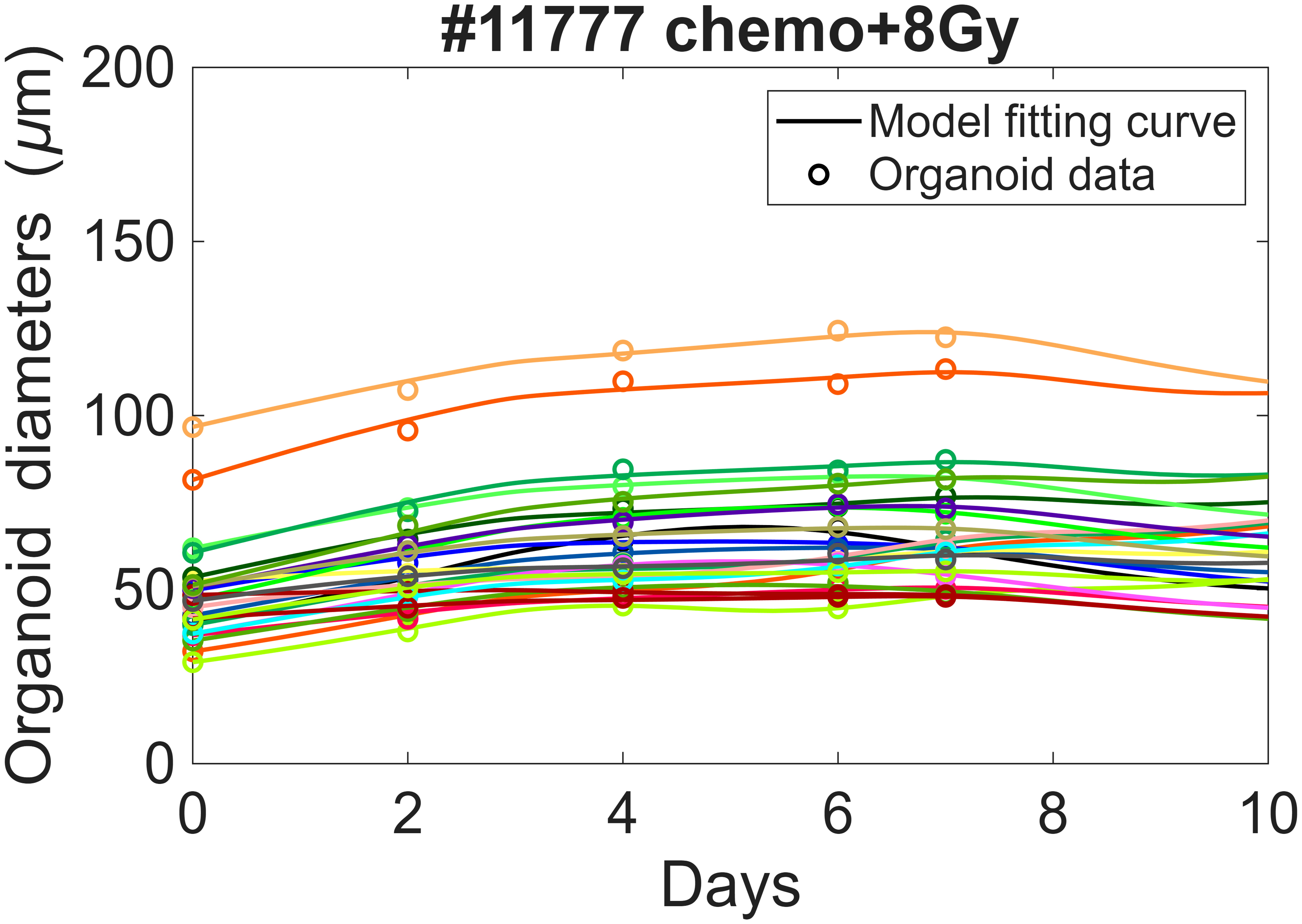


**E F**

**Figure S4.** Model curve fittings of organoids #11777 in each group. Solid lines are fitted curves, and circles of the same color represent data from one organoid collected on corresponding days. There are 30, 34, 35, 22, 23, and 25 organoid size data collected in the control, chemotherapy, radiotherapy with 4 Gy, radiotherapy with 8 Gy, chemoradiotherapy with 4 Gy, and chemoradiotherapy with 8 Gy groups, respectively. The data points were taken up to 7 or 9 days for each group.


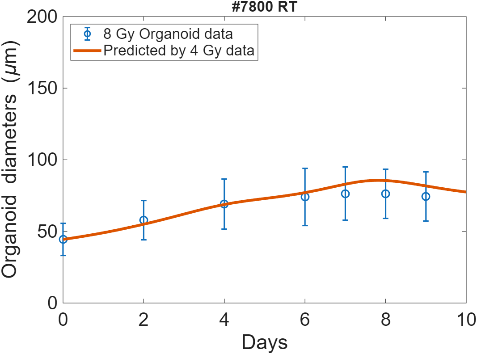

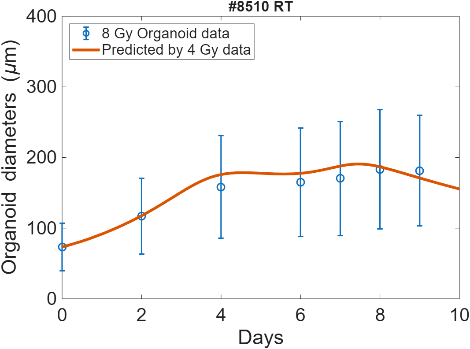

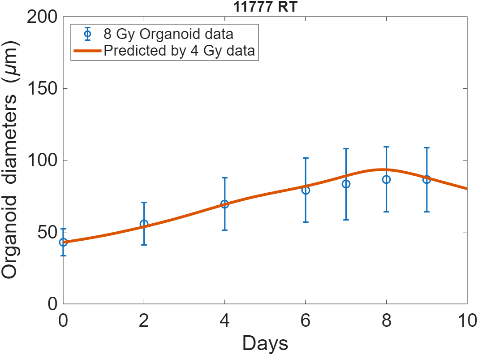


**Figure S5**: The blue circles with 95% CI are average data on corresponding days for 8 Gy experiments, and the solid orange curves are model predictions based on parameters obtained from 4 Gy experiments.


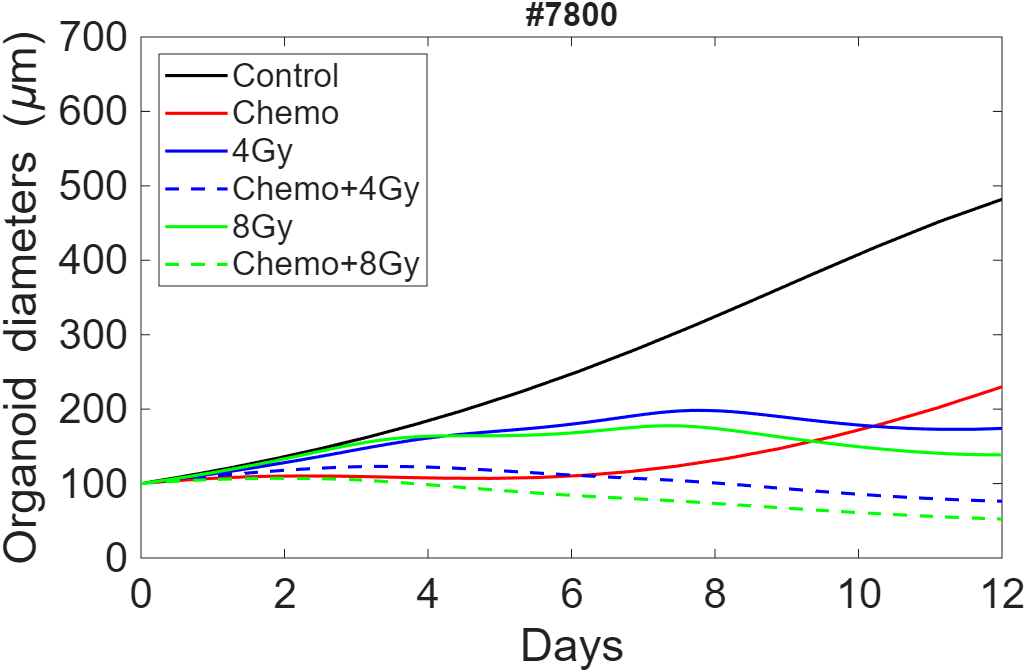

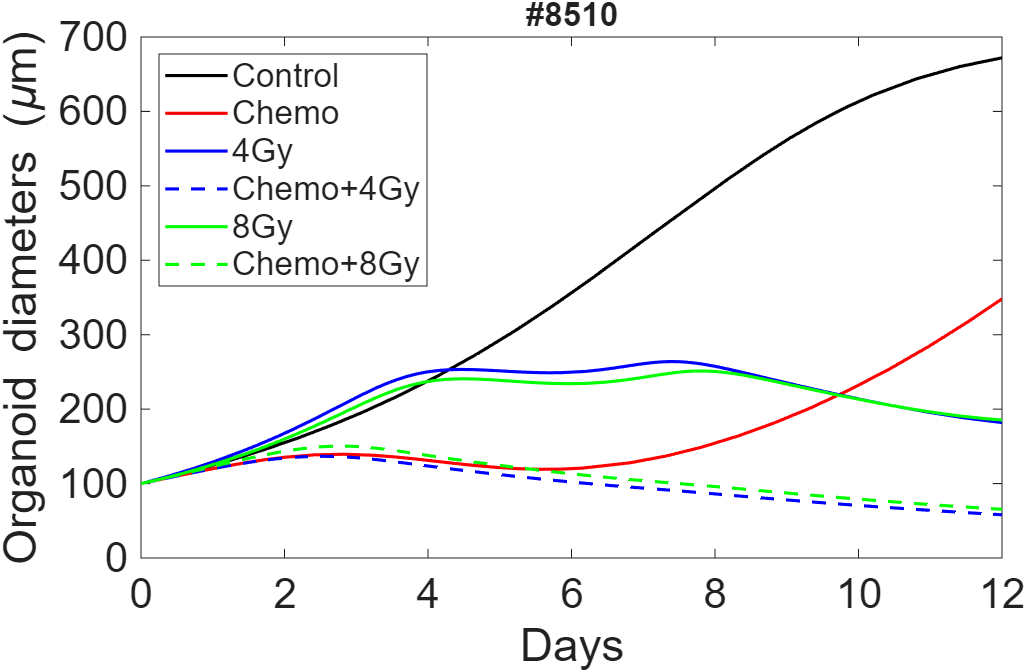

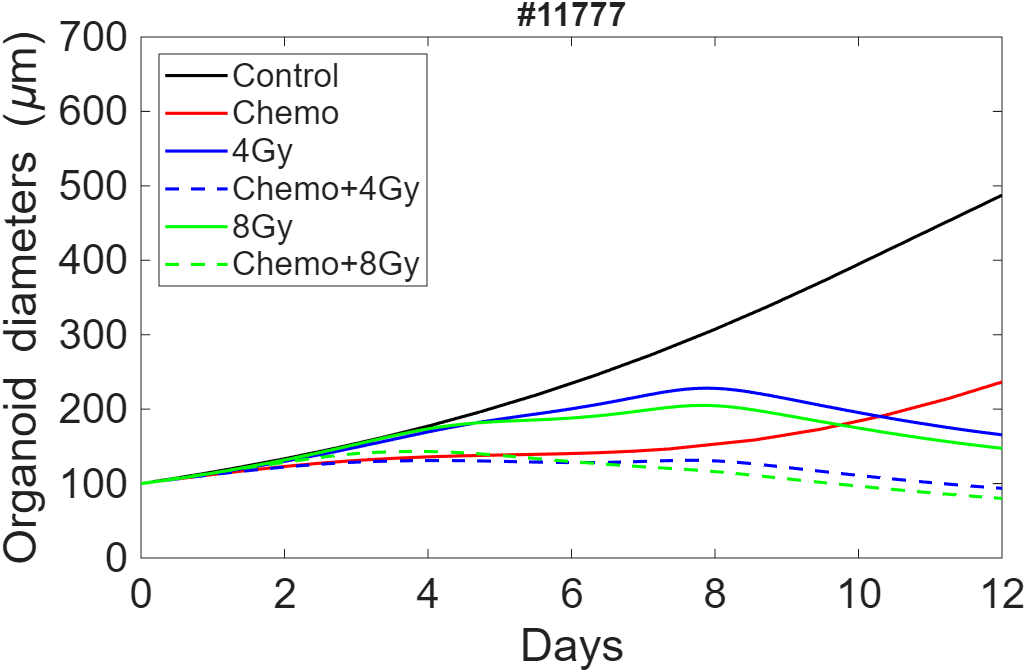


**A B C**

**Figure S6.** Simulated organoid diameters in each sample starting at 100 $\mu m$ change over time for up to 12 days in the scenarios of control, chemotherapy, radiotherapy at 4 Gy and 8 Gy, and chemoradiotherapy at 4 Gy and 8 Gy.

**Table S1.** Model predictions of treatment response (TR) values for each treatment on day 12.

| PDTOs ID | #7800 | #8510 | #11777 |
| --- | --- | --- | --- |
| Chemotherapy | 2.20 | 1.96 | 2.15 |
| Radiotherapy at 4 Gy | 2.98 | 3.75 | 3.20 |
| Radiotherapy at 8 Gy | 3.67 | 3.71 | 3.55 |
| Chemoradiotherapy at 4 Gy | 5.50 | 7.36 | 4.97 |
| Chemoradiotherapy at 8 Gy | 6.64 | 6.90 | 5.46 |

We compared our chemotherapy model (2) with model (S1) and radiotherapy model (3) with models (S2), (S3), and (S4), where models (S1) and (S2) have linear killing effects, models (S3) and (S4) are a single-compartment and a single-wave of radiation-induced killing effect model for radiotherapy, respectively.

$$\begin{aligned} \frac{dN}{dt}=\left( \lambda-a \right)N\left( 1-\frac{N}{K} \right), \#\left( S1 \right) \end{aligned}$$

$$\begin{aligned} \left\{ \begin{aligned} \frac{dA}{dt}=(\lambda-a)A\left( 1-\frac{A+I}{K} \right), \\ \frac{dI}{dt}=aA\left( 1-\frac{A+I}{K} \right)-\mu I, \end{aligned} \right.\#\left( S2 \right) \end{aligned}$$

where $a$ represents the killing strength.

$$\begin{aligned} \frac{dN}{dt}=(\lambda-R(t))N\left( 1-\frac{N}{K} \right). \#\left( S3 \right) \end{aligned}$$

$$\begin{aligned} \left\{ \begin{aligned} \frac{dA}{dt}=(\lambda-R_{1}(t))A\left( 1-\frac{A+I}{K} \right), \\ \frac{dI}{dt}=R_{1}(t)A\left( 1-\frac{A+I}{K} \right)-\mu I, \end{aligned} \right.\#\left( S4 \right) \end{aligned}$$

where $R_{1}\left( t \right)=u_{1}\exp\left( {-\left( t-T_{1} \right)}^{2}/{day}^{2} \right)$.

**Table S2.** Chemotherapy model comparison results using average AIC and BIC.

|  |  | Our Model (2) | Model (S1) |
| --- | --- | --- | --- |
| #7800 | AIC | 8.31 | 20.07 |
|  | BIC | 8.75 | 21.29 |
| #8510 | AIC | 15.31 | 35.51 |
|  | BIC | 15.74 | 36.72 |
| #11777 | AIC | 8.41 | 24.89 |
|  | BIC | 8.85 | 26.10 |

**Table S3.** Radiotherapy model comparison results using average AIC and BIC.

|  |  | Our Model (3) | Model (S2) | Model (S3) | Model (S4) |
| --- | --- | --- | --- | --- | --- |
| #7800 | AIC | 15.58 | 35.78 | 36.73 | 33.20 |
|  | BIC | 17.26 | 37.62 | 38.46 | 34.98 |
| #8510 | AIC | 31.54 | 58.22 | 54.16 | 50.43 |
|  | BIC | 33.22 | 60.05 | 55.89 | 52.22 |
| #11777 | AIC | 22.09 | 40.26 | 36.18 | 35.60 |
|  | BIC | 23.77 | 42.10 | 37.91 | 37.39 |

We performed additional experiments with the same PTDOs #7800, #8510, and #11777, following the same methods and procedures in Sections 2.2 and 2.3, except that no 8 Gy of radiotherapy and 8 Gy of chemoradiotherapy were administered. We measured 10 organoids from daily images for each PDTO line in control, chemotherapy, 4 Gy of radiotherapy, and 4 Gy of chemotherapy groups. The fitting results are presented in **Figures S7**, **S8**, and **S9**, and the comparison of NMSE values in **Table S2** demonstrates that our model does not significantly degrade the fit performance with more data and still provides a good fit.


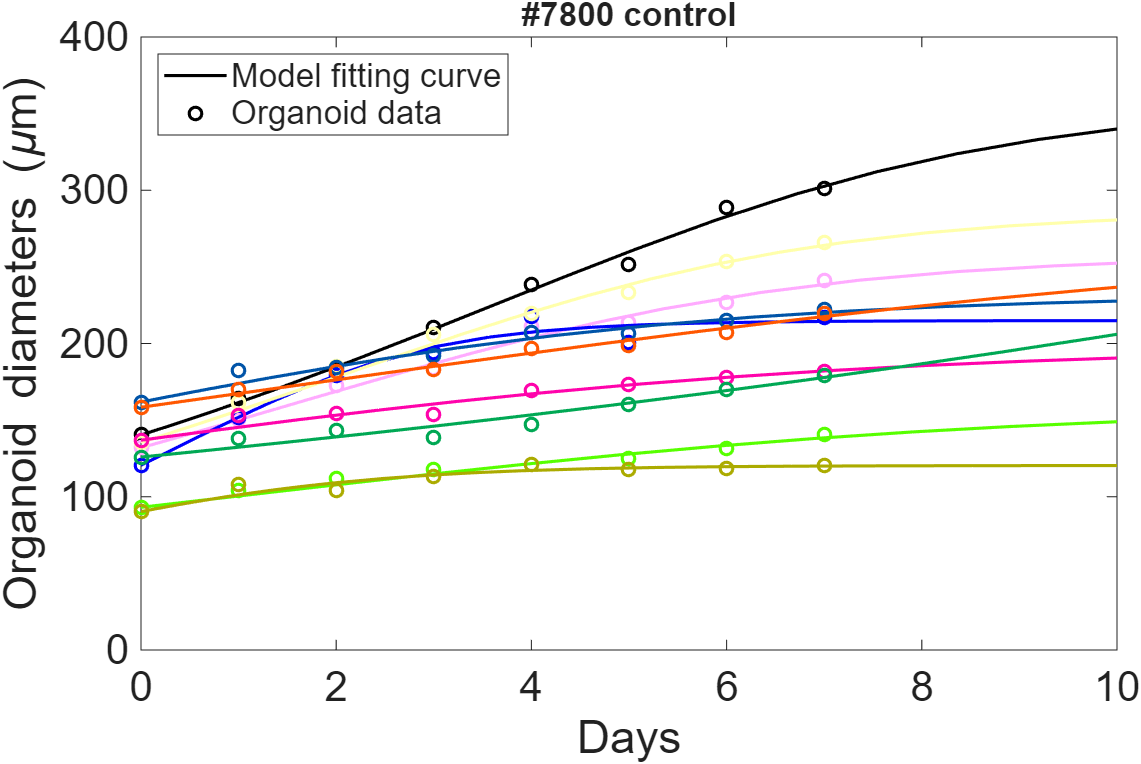

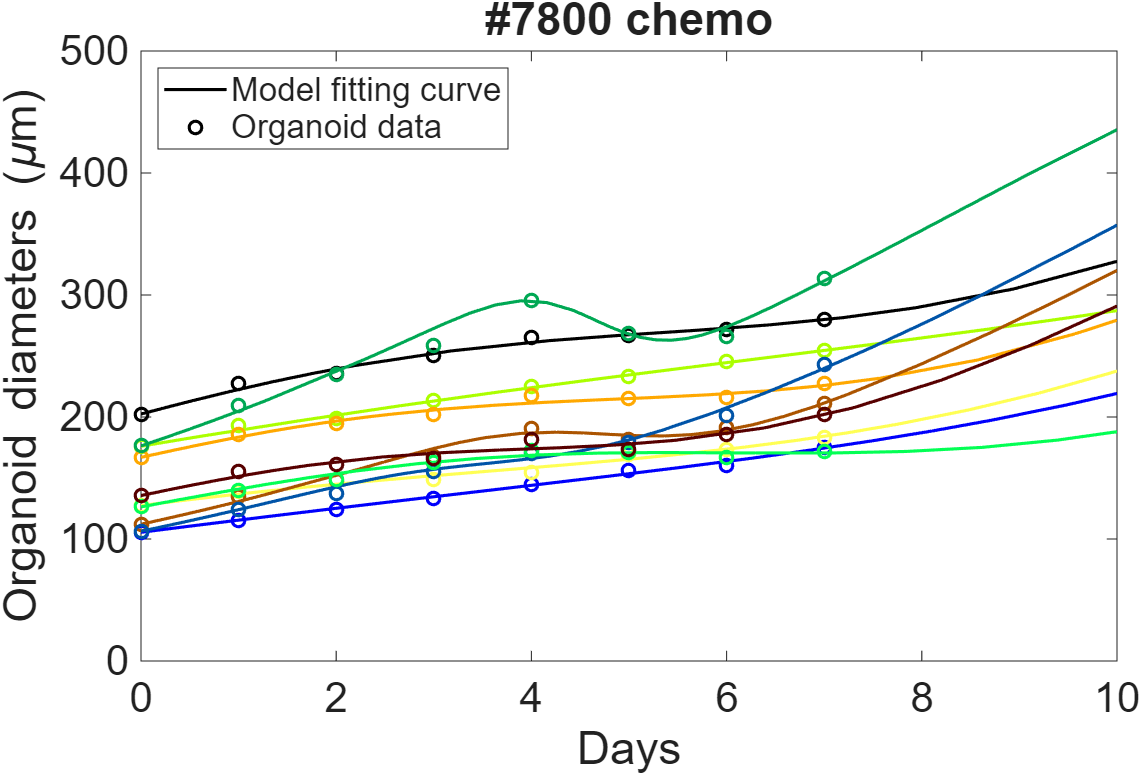


**A B**


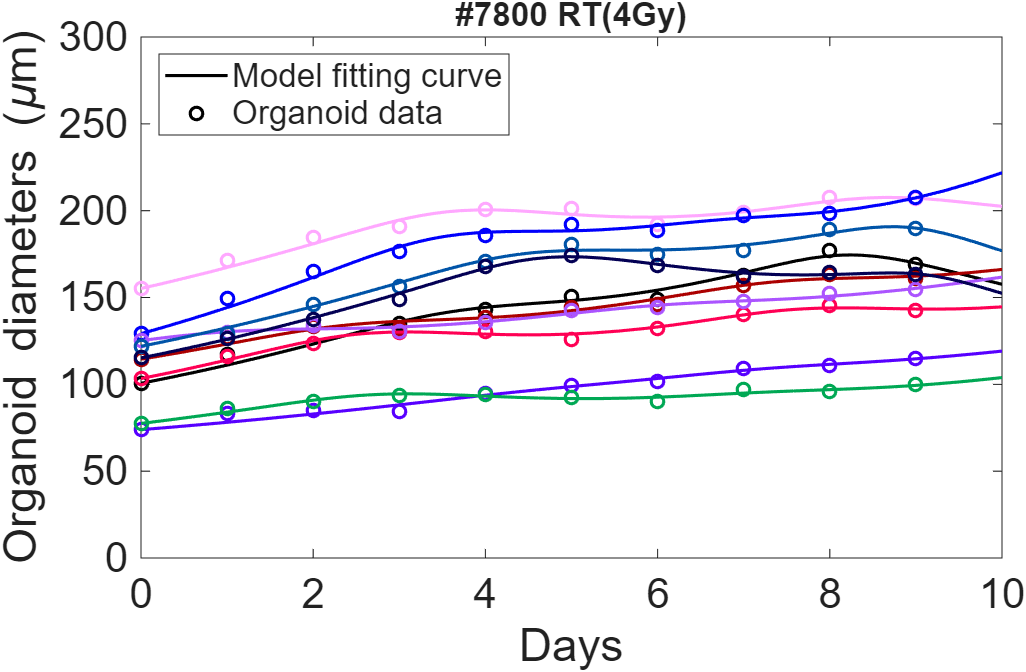

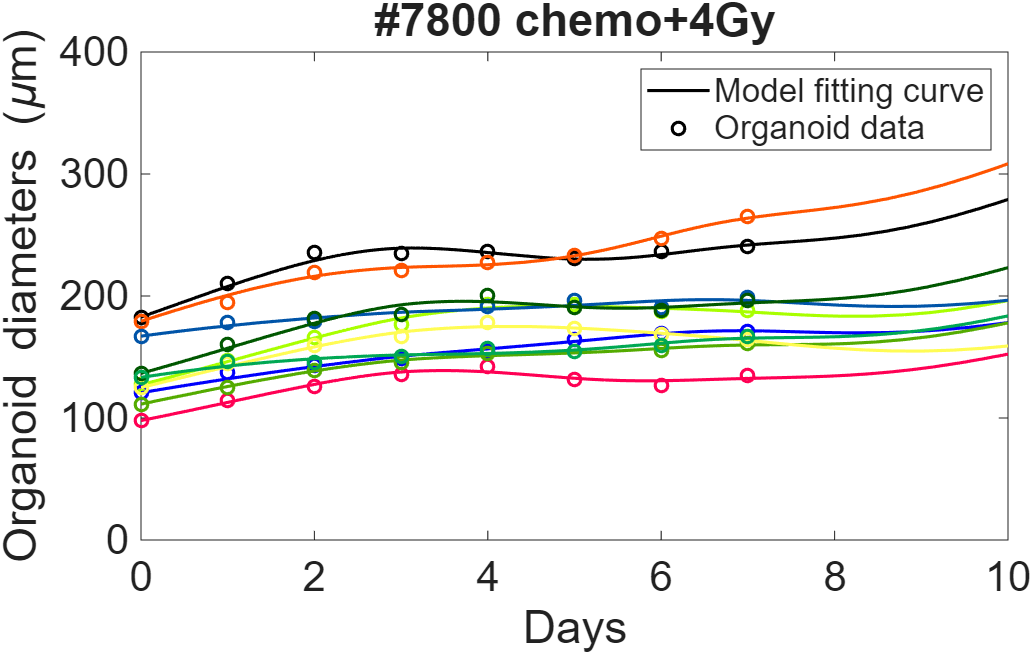


**C D**

**Figure S7.** An additional 10 fitted curves with data collected daily for organoids #7800 in the control, chemotherapy, radiotherapy, and chemoradiotherapy groups, with average NMSE values of 0.0037, 0.0024, 0.0016, and 0.0024, respectively.


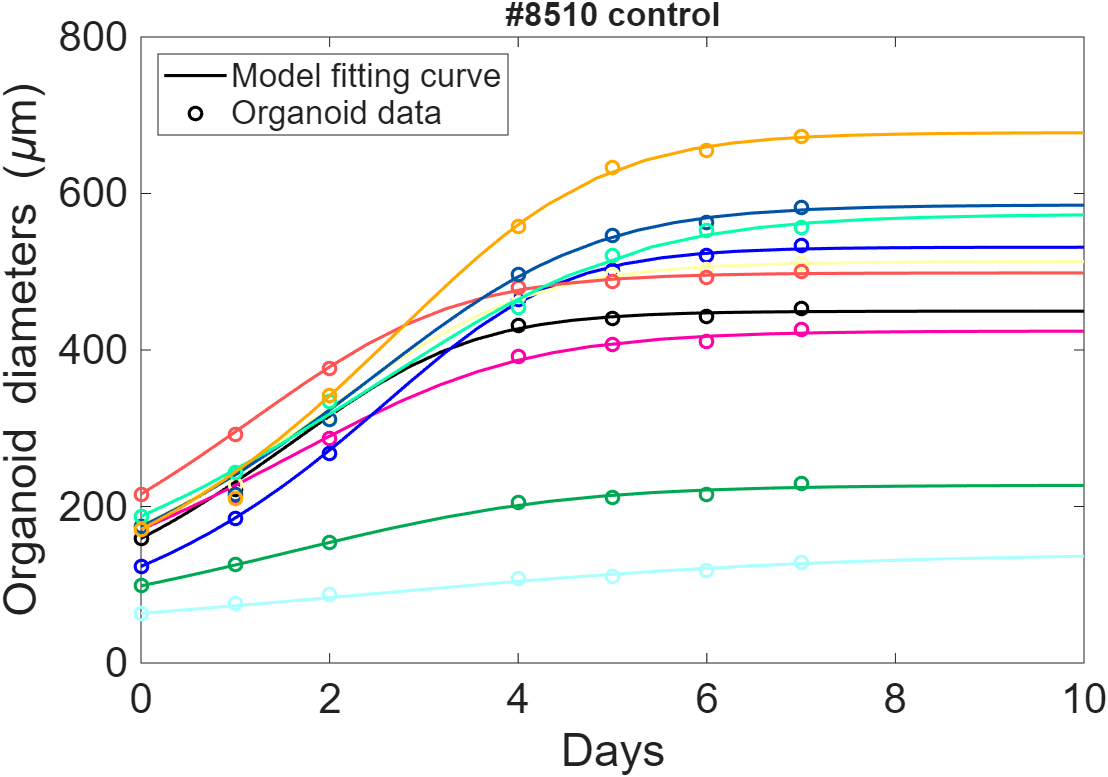

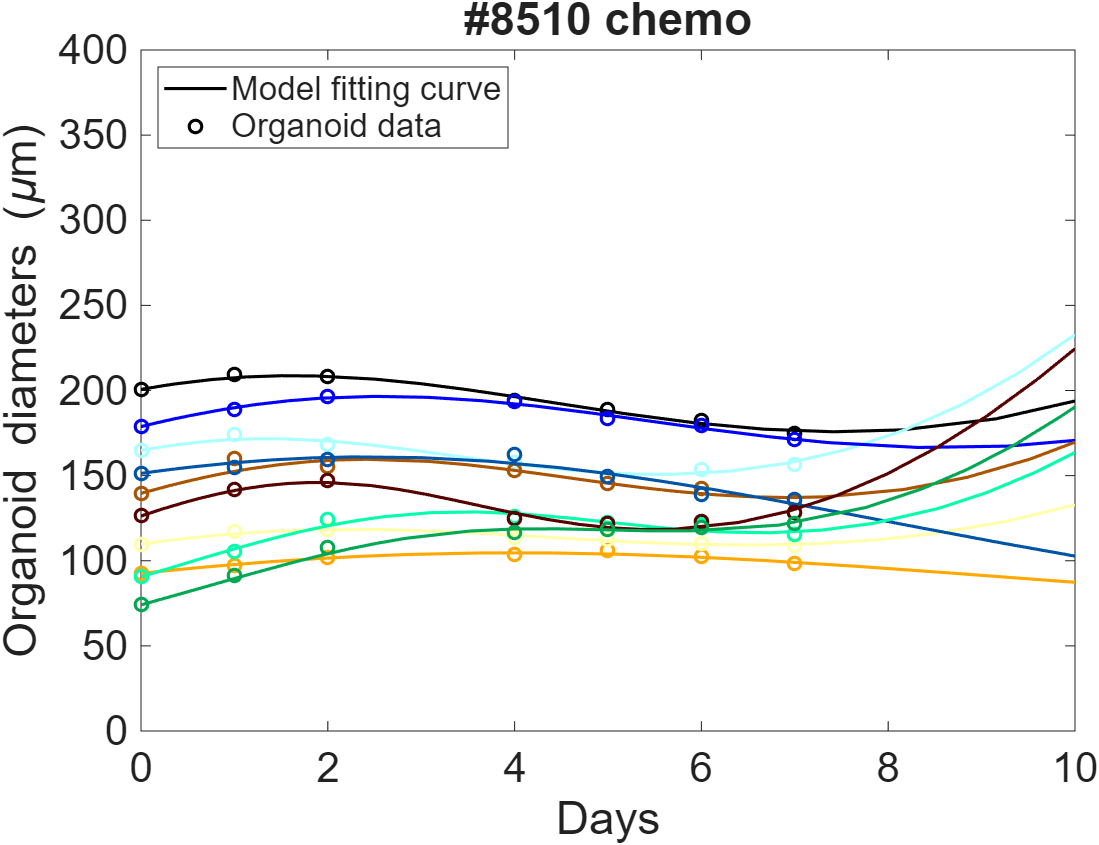


**A B**


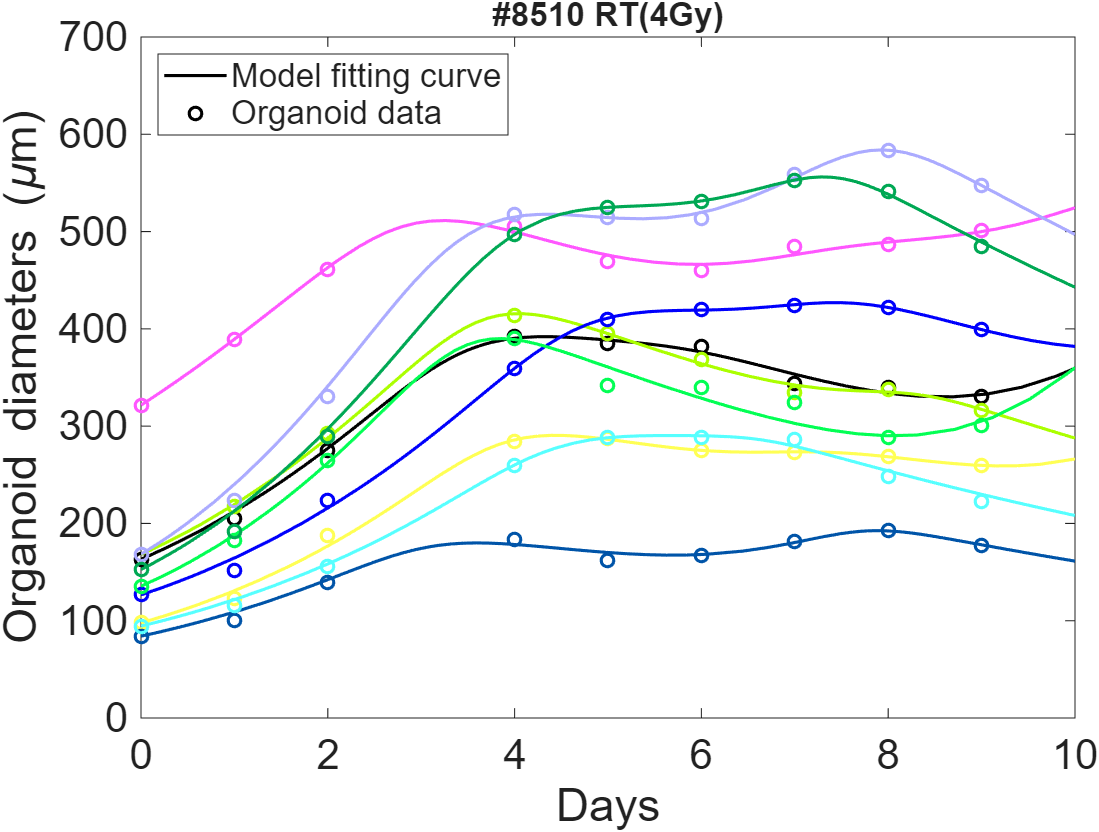

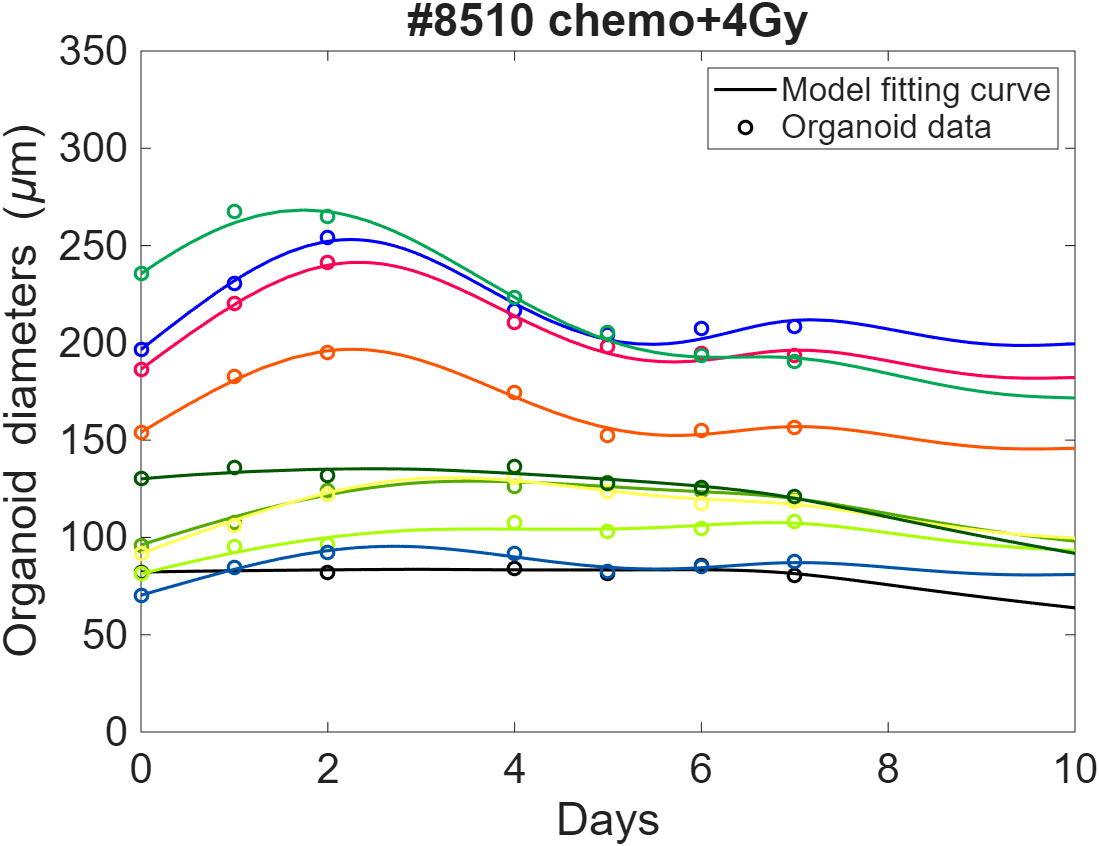


**C D**

**Figure S8.** An additional 10 fitted curves with data collected daily (except day 3) for organoids #8510 in the control, chemotherapy, radiotherapy, and chemoradiotherapy groups, with average NMSE values of 0.0026, 0.0020, 0.0017, and 0.0022, respectively.


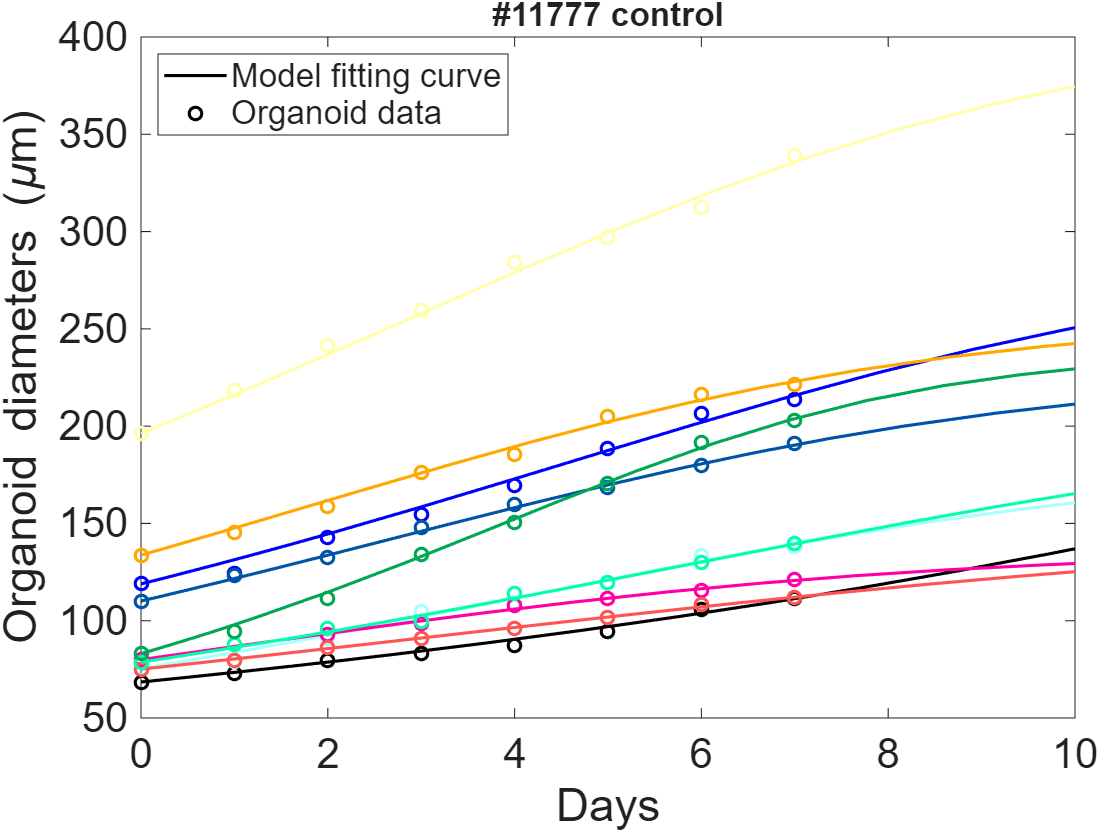

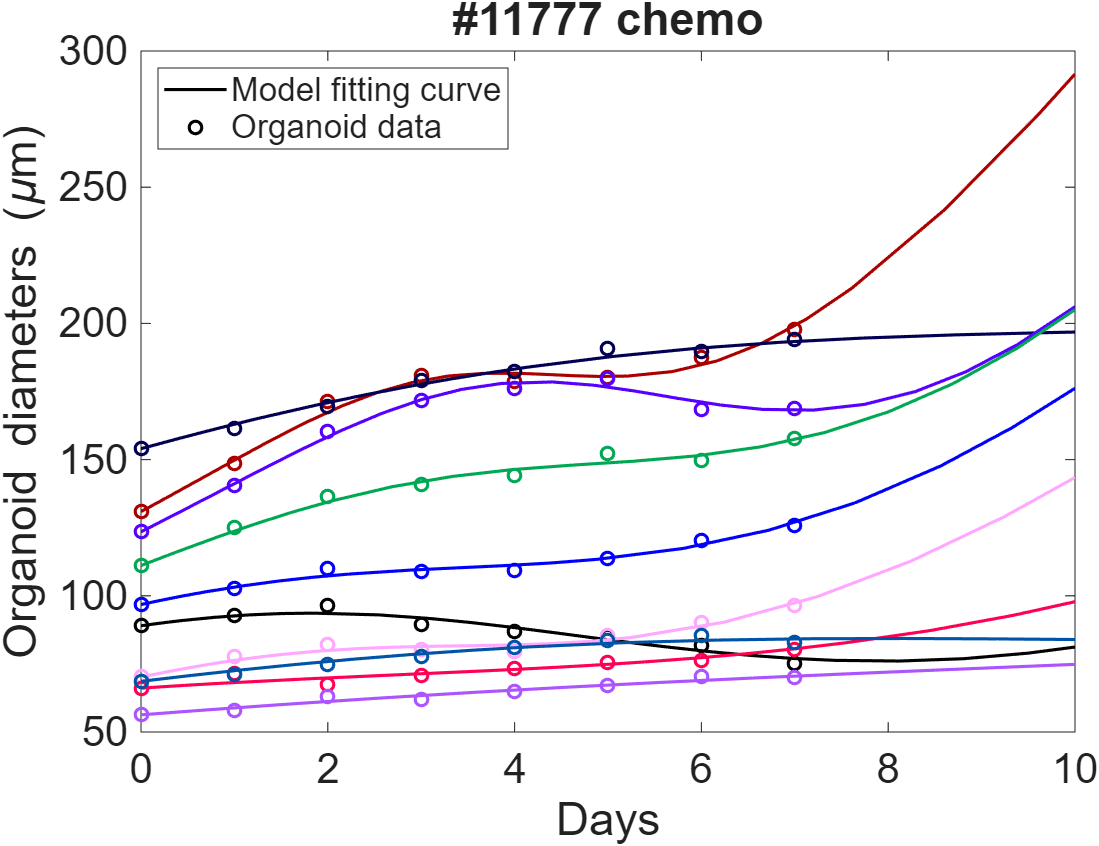


**A B**


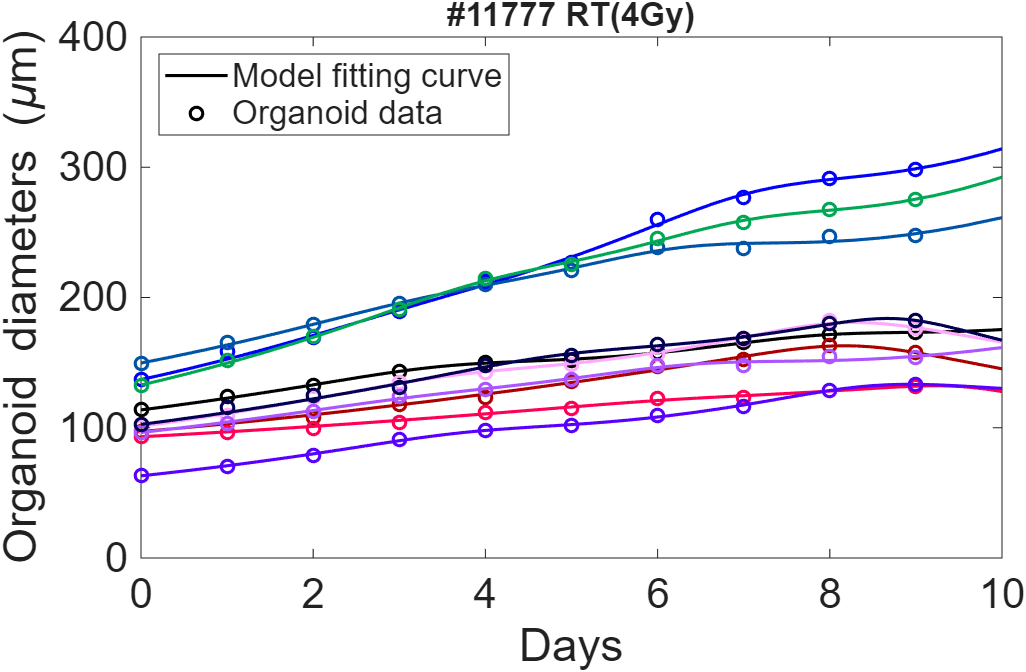

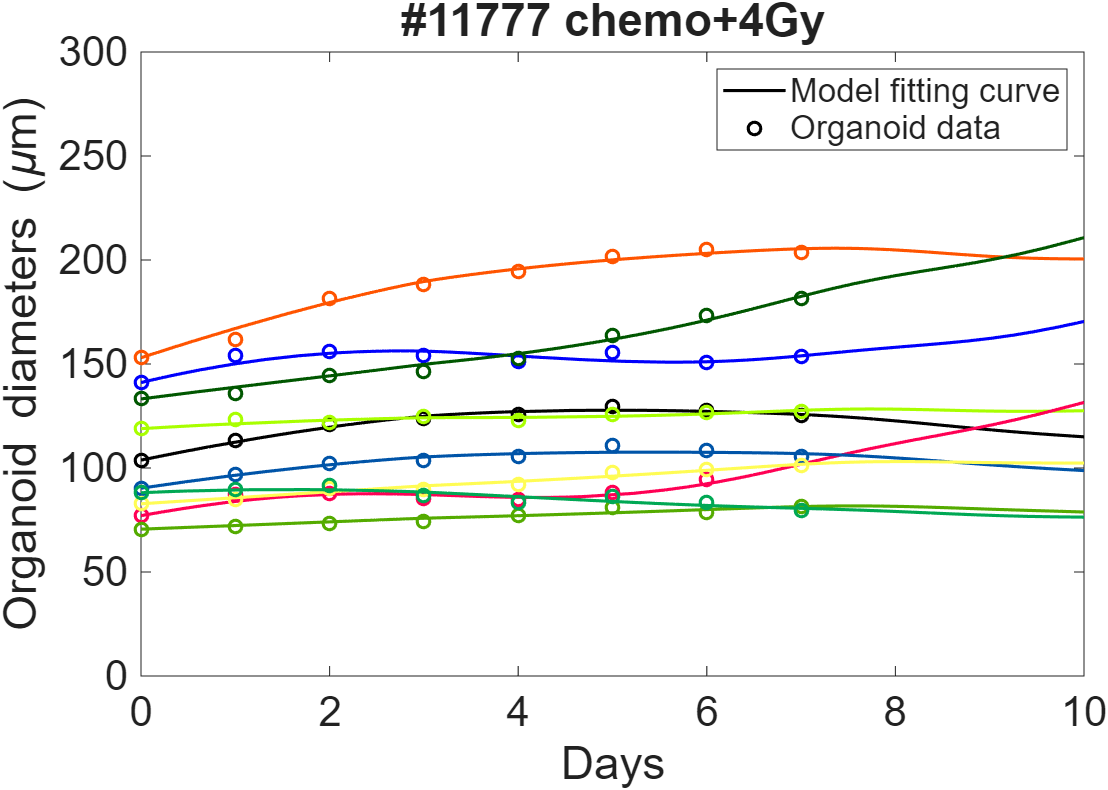


**C D**

**Figure S9.** An additional 10 fitted curves with data collected daily for organoids #11777 in the control, chemotherapy, radiotherapy, and chemoradiotherapy groups, with average NMSE values of 0.0019, 0.0020, 0.0007, and 0.0016, respectively.

In these two experiments, Exp.1 (initial) and Exp. 2 (additional), we compared the goodness-of-fit and treatment responses in **Tables S4** and **S5**. We can see that the average NMSE values remain on the same scale, and organoids #11777 show the lowest growth rate and organoids #8510 display the fastest growth rate and the largest maximum diameter; organoids #8510 are the most sensitive to chemotherapy and radiotherapy, whereas organoids #11777 exhibit the strongest drug resistance and the weakest early response, but a relatively stronger secondary response; the secondary-response are significantly higher than the early-response in radiotherapy for all organoids. These results further demonstrate the robustness of our models across different datasets.

**Table S4.** Comparison of the average NMSE values in the two experiments.

| PDTOs ID | #7800 | #8510 | #11777 |
| --- | --- | --- | --- |
| Control (Exp. 1) | 0.0028 | 0.0043 | 0.0017 |
| Control (Exp. 2) | 0.0037 | 0.0023 | 0.0016 |
| Chemotherapy (Exp. 1) | 0.0010 | 0.0022 | 0.0012 |
| Chemotherapy (Exp. 2) | 0.0023 | 0.0018 | 0.0023 |
| Radiotherapy at 4 Gy (Exp. 1) | 0.0004 | 0.0025 | 0.0009 |
| Radiotherapy at 4 Gy (Exp. 2) | 0.0016 | 0.0017 | 0.0007 |
| Chemoradiotherapy at 4 Gy (Exp. 1) | 0.0017 | 0.0022 | 0.0024 |
| Chemoradiotherapy at 4 Gy (Exp. 2) | 0.0024 | 0.0022 | 0.0016 |

**Table S5.** Comparison of the treatment responses in two experiments.

|  |  |  | #7800 | #8510 | #11777 |
| --- | --- | --- | --- | --- | --- |
| Control | $\lambda$ (day ^-1^) | Exp.1 | 0.464 ± 0.033 | 0.657 ± 0.053 | 0.431 ± 0.047 |
|  |  | Exp. 2 | 0.430 ± 0.168 | 0.910 ± 0.141 | 0.340 ± 0.060 |
|  | $D_{K}$ ($\mu m$) | Exp.1 | 302.8 ± 155.2 | 406.5 ± 164.2 | 360.8 ± 0.047 |
|  |  | Exp.2 | 356.5 ± 283.5 | 461.8 ± 115.7 | 349.2 ± 281.1 |
| Chemotherapy | $a$ (day ^-1^) | Exp.1 | 0.515 ± 0.037 | 0.907 ± 0.123 | 0.392 ± 0.026 |
|  |  | Exp.2 | 0.426 ± 0.150 | 1.058 ± 0.053 | 0.356 ± 0.057 |
| Radiotherapy | $u_{1}$ (day ^-1^) | Exp.1 | 0.486 ± 0.090 | 1.699 ± 0.204 | 0.373 ± 0.090 |
| at 4 Gy |  | Exp.2 | 0.658 ±0.198 | 1.880 ± 0.133 | 0.283 ± 0.119 |
|  | $u_{2}$ (day ^-1^) | Exp.1 | 1.518 ± 0.693 | 3.726 ± 0.545 | 2.695 ± 0.738 |
|  |  | Exp.2 | 1.369 ± 1.211 | 3.528 ± 1.557 | 2.004 ± 1.574 |
| Chemoradiotherapy | $a$ (day ^-1^) | Exp. 1 | 0.482 ± 0.042 | 0.939 ± 0.115 | 0.374 ± 0.032 |
| at 4 Gy |  | Exp. 2 | 0.428 ± 0.059 | 1.080 ± 0.111 | 0.328 ± 0.045 |
